# Epidemic predictions in an imperfect world: modelling disease spread with partial data

**DOI:** 10.1098/rspb.2015.0205

**Published:** 2015-06-07

**Authors:** Peter M. Dawson, Marleen Werkman, Ellen Brooks-Pollock, Michael J. Tildesley

**Affiliations:** 1 Centre for Complexity Science, University of Warwick, Coventry CV4 7AL, UK; 2 School of Veterinary Medicine and Science, University of Nottingham, Sutton Bonington LE12 5RD, UK; 3 Central Veterinary Institute, Wageningen UR (CVI), PO Box 65, 8200 AB Lelystad, The Netherlands; 4 School of Social and Community Medicine, University of Bristol, Bristol BS8 2BN, UK; 5 Fogarty International Center, US National Institute of Health, Bethesda, MD 20892, USA

**Keywords:** epidemics, livestock networks, partial data

## Abstract

‘Big-data’ epidemic models are being increasingly used to influence government policy to help with control and eradication of infectious diseases. In the case of livestock, detailed movement records have been used to parametrize realistic transmission models. While livestock movement data are readily available in the UK and other countries in the EU, in many countries around the world, such detailed data are not available. By using a comprehensive database of the UK cattle trade network, we implement various sampling strategies to determine the quantity of network data required to give accurate epidemiological predictions. It is found that by targeting nodes with the highest number of movements, accurate predictions on the size and spatial spread of epidemics can be made. This work has implications for countries such as the USA, where access to data is limited, and developing countries that may lack the resources to collect a full dataset on livestock movements.

## 1. Introduction

Modelling of infectious diseases is a rapidly growing field in which mathematical modellers can play a significant role in determining how applied knowledge can be translated into an understanding of dynamics at the population level [1–4]. During the foot-and-mouth disease (FMD) outbreak in the UK in 2001, several research groups developed a range of models that were able to predict the spatio-temporal pattern of disease spread and the impact of control strategies [1,3,5]. The use of these models in 2001 highlighted the role that models could play in shaping policy. Since then, the infection data from the 2001 epidemic have enabled research to be carried out to predict optimal culling and vaccination strategies both for the 2001 epidemic itself and for any future FMD epidemic in the UK and elsewhere [6–9].

In the 2001 FMD outbreak, early dissemination of the disease, prior to the first detected case, was mainly a result of long-distance movement of livestock between farms and through markets [10]. In other countries where FMD is endemic, livestock movements are believed to play a significant role in disease persistence [11]. Movements of live animals are also thought to cause significant transmission of diseases such as bovine tuberculosis (bTB) in the UK [12,13], as well as vector-borne diseases such as trypanosomiasis in southeast Asia, Australasia and Africa [14,15], and Rift Valley fever in Africa [16]. In cases such as these, infected livestock may be moved prior to showing symptoms, and therefore there is a risk of long-distance spread occurring. It is therefore crucial to understand the risk of infection spread associated with livestock movements.

In the UK, an annual livestock census records the location and species composition of all livestock farms. Births, deaths and movements of animals are recorded for individual cattle via the Cattle Tracing System (CTS), and for batches of other livestock via the Animal Movement Licence scheme. Such data have driven the development of sophisticated models to capture and predict the spread of livestock diseases such as FMD [1,8,17,18], bovine tuberculosis [12,19] and *Escherichia coli* [20]. However, many countries around the world do not routinely collect farm-level data, or they are not readily available for research owing to issues regarding privacy. For example, in the USA, the National Agricultural Statistics Service carries out an agricultural census every 5 years. In order to preserve anonymity for farmers, all data are aggregated at the county level, and therefore precise locations of livestock farms are unknown. Furthermore, movement data are held at the individual state level, and there is no requirement for livestock movements to be recorded unless movements are out of state [21]. In the UK, the poultry industry infers movements between holdings using targeted sampling of premises based upon their function and size. This method predominantly targets large farms, and therefore does not accurately capture the demographic characteristics of the underlying farm population [22]. Therefore, it is important to understand the ability of models to predict the potential for disease spread through livestock movements when only a partial sample of the network is available.

Partial network data constitute a well-known problem, and have been studied extensively in social sciences and other fields, such as epidemiology in humans and livestock diseases. While the amount of data available to modellers is increasing, so too are privacy concerns. In order to predict the risk of disease spread in humans across large spatial scales, detailed movement networks must be established. These networks can be informed using commuting and migration data available from population censuses [23–25]. These data capture long-term trends, but may not be appropriate at predicting movements over a shorter time scale, and therefore can be complemented by the inclusion of other information such as mobile phone data. Mobile phone records track locations and times that individuals make and receive calls, and therefore can act as a proxy for shorter-scale movement patterns [23,26]. While full access to these datasets is not readily available, previous work indicates that partial samples may be sufficient to accurately predict the risk associated with disease spread across these networks [24,27].

In situations where only partial network information is available, it may be necessary to reconstruct the network. Different approaches can be applied to construct contact networks. The most basic methods involve random sampling of nodes (i.e. individuals or farms [28]) or random sampling of edges (i.e. links between nodes). However, it may be possible to capture the key properties of a network more efficiently using an approach such as snowball sampling (SBS). SBS is typically used in situations where the target population is small and hard to find. A number of individuals from the target population are asked to nominate an *x* number of people from the target population [29]. This method has been used previously to identify networks of sexual contacts for HIV-positive individuals [30]. In the case of livestock, when there may be knowledge regarding the size of farms or number of aggregated movements from a farm (per year), a targeted sampling approach could be used where larger farms or farms with the highest number of movements are sampled. When constructing networks, including specific network characteristics (such as age structure) improves the quality of the constructed network [31]. Previous studies have shown that subsets of networks are not always representative of the whole network (e.g. scale-free networks [28]). Therefore, caution is needed when networks are constructed with partial data.

In previous work, Tildesley *et al.* [32] demonstrated that even in the absence of precise locations of farms, accurate predictions of the impact of interventions are possible. We aim to develop an understanding of the predictive power of mathematical models when only a subset of the network information is available. We develop a model to simulate the spread of a rapidly spreading disease such as FMD through the UK cattle movement network. Mathematical models have previously played a key role in determining the risk of disease spread through networks of livestock movements for diseases such as FMD [17,18,21,33], bTB [12,13,19] and bluetongue virus [34,35]. Our aim in this paper is to investigate the ability of such models to provide policy advice in countries where only partial information regarding livestock movements is available.

We compare four imperfect data types: random sampling of movements (weighted edges), random sampling of farms (nodes), SBS [30,36] of farms and targeted sampling of farms. If appropriate, we then scale the sampled networks up, so that the original number of movements is used for the epidemic simulations. In the UK, selling and buying of livestock often takes place through livestock markets. Previous work suggests that these markets played a substantial role during the 2001 FMD outbreak [10]. As animals from different farms are kept in close proximity, there is a risk of disease transmission between batches of animals resulting in spread of infection to multiple farms. Moreover, it is known that movements from markets cover a large geographical area [37]. Therefore, we investigate the potential role of markets in disease transmission between farms.

This study will be highly informative for countries where livestock movement data are not routinely available. The outputs of this work will provide guidance to livestock industries around the world regarding both the quantity of data required to predict spread of disease and how to target data collection should it not be possible to record all livestock movements.

## 2. Material and methods

In this paper, we use data from the 2010 CTS database for Great Britain, provided by the Department for the Environment, Food and Rural Affairs (Defra) via the Animal and Plant Health Agency. If multiple animals were moved on the same day from one farm to another, this was treated as one movement; markets were initially not explicitly included. Slaughterhouses were considered as sinks, and therefore movements to slaughterhouses were ignored even when going through a livestock market. In total, there were 70 243 farms and 327 markets in our dataset, with 856 454 movements in total. A total of 635 016 movements passed through markets, with 47 692 farms using cattle markets at least once during 2010.

Four methods of sampling from this database are implemented and compared here. A directed weighted-static adjacency matrix *A* [38] was constructed for each set of sampled data, in which nodes represent farms and edges represent (directed) cattle movements. An edge *a_ij_* is defined to be non-zero if cattle are moved from farm *i* to farm *j* during the year. The weight of the edge represents the frequency of movements from farm *i* to farm *j* in 2010 (i.e. the total number of days on which movements occurred divided by 365).

### (a) Movement sampling

For random movement sampling (RMS), we list the recorded movements and randomly sample from this list. The depleted network is then built from the remaining movements and the resultant network is rescaled such that the total weight of the rebuilt network is equal to that of the original network,2.1
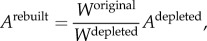
where 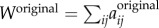 and 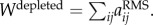 This method explicitly depends on knowledge of the total weight of the network.

### (b) Node sampling

In the node sampling schemes, a ‘sampled’ node has all its edges sampled. A node is said to be ‘captured’ if it is connected to a sampled node but it has not been sampled itself. For all of these schemes, we assume that the total number of nodes *N* in the network is known. We sample a set *S* of *N*_S_ nodes and capture *N*_C_ nodes, the *N*_S_ sampled nodes plus their connected non-sampled neighbours. This method will therefore preserve the degree of the initially sampled nodes *N*_S_, but for the remaining nodes that are captured, only the edges that link them to the *N*_S_ nodes will be recorded.

We consider three methods of node sampling in this paper. In the first method, we use random node sampling (RNS) such that all movements from a certain percentage of nodes are selected. A more advanced form of node sampling is SBS [36]. In this method, an initial set of nodes are sampled at random. At the next stage, the nodes captured by the initial sampled nodes are in turn sampled. This process can continue until all nodes have been sampled. In this paper, we consider second-order SBS, such that an initial set of nodes are sampled and the nodes that this set of nodes are connected to are also sampled.

The final node sampling scheme considered is targeted node sampling (TNS). In TNS, we sample specific nodes based on certain criteria. In this case, we chose the weighted-degree of the node. All nodes having a weighted-degree of at least *x* are sampled and their neighbours are captured (electronic supplementary material, figure S1). A graphical depiction of the three node sampling network schemes is shown in the electronic supplementary material, figure S2.

We first considered rescaling the network formed by the node sampling methods in a similar way to that for the RMS method. We used the average weighted-degree of the sampled nodes 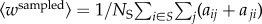 to estimate the total weight of the original network 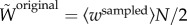, and rescale the network as in equation (2.1) but using the estimate for the total weight of the original network. However, as shown in electronic supplementary material, figure S6, these scaled networks result in significant overpredictions of epidemic size, particularly when small percentages of the nodes are sampled. For the remainder of this paper, we therefore use the unscaled versions of the node sampling methods.

### (c) Network statistics

The properties of the underlying network may have a significant effect on epidemic dynamics [39]. We therefore consider how network properties change as fewer data are used by the various sampling schemes. We first consider the number of strongly connected components of the network. A subset of nodes forms a strongly connected component if each of the nodes can connect to each other node by following a path which preserves edge direction. If the largest of these components is of the same order as the complete network it is known as the giant strongly connected component (GSCC) and gives a lower bound to the maximum size of an outbreak on the network if the disease is perfectly transmissible [33]. In addition, we explore the impact of the sampling schemes upon the mean and standard deviation of the weighted *w* and unweighted *k* degree of nodes in the GSCC. Finally, we investigate the tendency for similar nodes to connect to each other through degree assortativity [40,41]. These statistics are averaged over 1000 realizations of the network for each sampling method. The diameter of the GSCC was also measured but, owing to extensive computational time, this was only calculated for a single realization of the network. The diameter of a network is the length of the longest shortest path across the network [40]. As well as the network statistics mentioned previously, we also explore the number of nodes and edges captured by the various sampling schemes as the percentage of sampled data varies.

### (d) Comparison of epidemic predictions

A stochastic susceptible–infectious–recovered (SIR) model was used to investigate epidemic behaviour on the livestock network. The probability of farm *i* becoming infected is defined as2.2
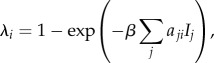
where *I_i_* = 1 if farm *i* is infected and zero otherwise, and *β* is the transmission rate. Infected farms recover after a period *T* and cannot be reinfected.

We aim to investigate spread of relatively ‘fast-moving’ diseases in the absence of movement restrictions such as FMD. We make the assumption that transmission of infection to a farm results in all animals on that farm moving into the infectious class. Given this assumption, the risk of infection between any pair of nodes in the movement network is based upon the number of movements between them rather than the number of animals moved. In order to investigate the impact of epidemiological parameters upon model predictions, we explore a range of values for the transmission parameter and the infectious period, such that *β* = 1, 2, 5, 10 and *T* = 7, 14, 21, 28 days.

After reconstructing the movement network, epidemics were seeded randomly in Cumbria, Aberdeenshire or Devon. These three counties have a high number of cattle farms and livestock movements, and therefore epidemics starting in these counties are more likely to produce a high number of cases than in other parts of the UK. Cumbria and Devon were also two major hotspots of infection during the UK FMD outbreak in 2001 [3]. A random source farm in each county was infected initially for each simulation, and we investigated the predicted final epidemic size, duration, peak size and the model prediction of the geographical spread of disease.

A thousand networks were created for each sampling scheme. Of these, 100 were randomly selected for simulations. Statistics are averaged over 1000 simulations that had a final epidemic size of at least 10 farms. Pseudo-code for the SIR process is included in electronic supplementary material, algorithm 1.

### (e) Livestock markets

Markets may play a key role in amplification of disease transmission [37]. The CTS explicitly states whether a movement went through a market and, if so, which market was used. This allows us to construct networks that include markets as nodes. The above-listed sampling schemes can all be applied to this situation. There is significant uncertainty regarding the level of contact of animals from different batches (farms) on a market, and therefore the risk of transmission between animals during their stay on the market. For this reason, we investigate the effect of two extreme assumptions of transmission within a market (electronic supplementary material, figure S3). In the first scenario, we assume no within-market transmission, such that infection is only transmitted between the source and the destination farm. We assume complete segregation between herds being strictly enforced (this would be equivalent to having no markets in the network). In the second scenario, we assume no segregation and no biosecurity at a market, such that all batches that move through a market mix with one another homogeneously. In this case, we use the CTS data to determine which batches of cattle move through a market. When an infected batch moves to a market, that market becomes infected and we then assume that infection can be transmitted to all possible destination farms (as determined based on the destinations of all batches that move from the market) with an equal probability. A graphic depicting how the network is altered by the inclusion of markets is shown in the electronic supplementary material, figure S3, and pseudo-code for the updated epidemic process is shown in the electronic supplementary material, algorithm 2.

In the UK, livestock has to be removed 4 h after the last market sale and consequently does not stay overnight at a livestock market [42]. Therefore, we assumed that cattle are moved on and off a market on the same day, and that an infectious market becomes susceptible again the following day. If this assumption were to be relaxed, the model could be altered by giving markets a longer infectious period.

Simulations are carried out in the same way as detailed in §2d with the one exception that we only run outbreaks for the length of one infectious period. The increased transmissibility from the inclusion of markets results in substantially larger epidemics, and therefore one infectious cycle is sufficient to analyse the effects of the different sampling methods.

## 3. Results

### (a) Comparison of network statistics

**Figure 1.**
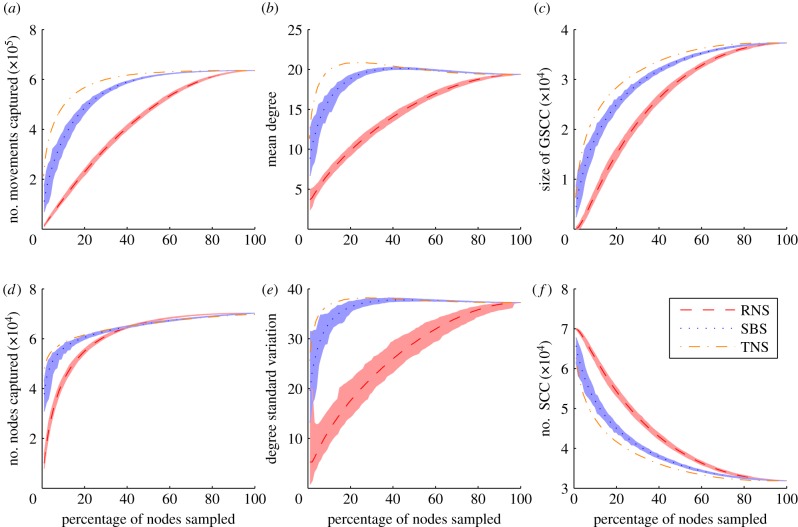
(*a*) The number of movements captured, (*b*) the mean degree, (*c*) the size of the giant strongly connected component, (*d*) the number of nodes captured, (*e*) the degree standard deviation and (*f*) the number of strongly connected components for the RNS (dashed line), SBS (dotted line) and TNS (dotted-dashed line) as a function of the percentage of nodes sampled. These statistics are averaged over 1000 realizations of the network for RNS and SBS with shaded confidence intervals (CIs) depicting the maximum and minimum value of each statistic.

As the percentage of nodes sampled decreases, the number of movements and nodes captured is observed to decrease for all sampling methods (figure 1). As the network fragments, the size of the GSCC decreases while the number of strongly connected components increases. Both the TNS and SBS sampling schemes outperform the RNS scheme in preserving robustness across all measured statistics as the percentage of nodes sampled decreases (figure 1). Within the giant component, the mean degree and degree standard deviation remain robust with approximately 15–20% of the data for both TNS and SBS.

The complete network has assortativity coefficients close to zero, meaning there is not a tendency for similar nodes to connect to or avoid each other. This holds true within the GSCC for all node sampling schemes (electronic supplementary material, figure S4). Similar behaviour is observed for the mean local clustering coefficient, which is small and does not change appreciably. The network diameter is 24—TNS and SBS preserve this relatively small diameter well within the GSCC, but for small sample sizes the diameter increases under the RNS scheme. Plots for assortativity, clustering and diameter are shown in electronic supplementary material, figure S4.

### (b) Comparison of epidemic predictions

In order to explore the epidemiological effects of the various sampling methods, we compare each method with simulations run on the full network. The robustness of a sampling method is determined by whether the mean simulation for a method using a certain percentage of data lies within the 95% CIs of the mean of simulations run in the full network. We focus on key epidemiological quantities such as final size, peak size and epidemic duration. While it is informative to explore the effect of partial knowledge upon epidemic duration, for many diseases livestock movement bans will be implemented as soon as cases are reported. We therefore also look at predictions of the epidemic size after 6 and 12 weeks using the different sampling methods. We denote the threshold at which a scheme fails to be robust as *S*_min_, the minimum sampling threshold. Initially, we set *β* = 1 and *T* = 21 days. Sensitivity to these parameter values is explored below (electronic supplementary material, figures S13–S16).

**Figure 2.**
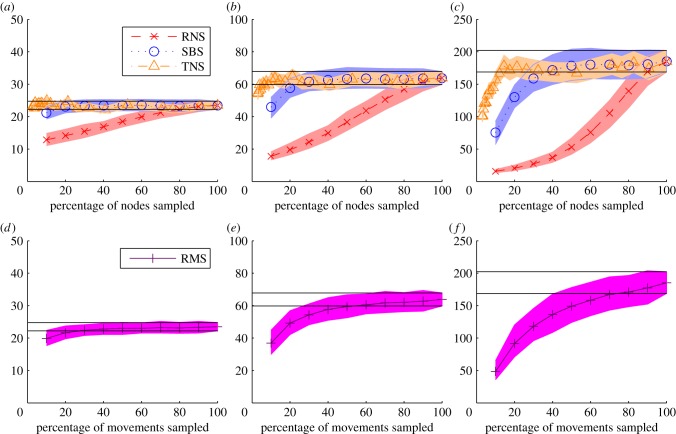
(*a*–*c*) Epidemic size for outbreaks seeded in Cumbria on networks generated by RNS (crosses), SBS (circles) and TNS (triangles) as a function of nodes sampled with shaded 95% CIs for (*a*) 6 weeks, (*b*) 12 weeks and (*c*) the full epidemic. The solid black lines represent the 95% CIs on the average simulation for the original network. (*d*–*f*) The same results for the RMS method for (*d*) 6 weeks, (*e*) 12 weeks and (*f*) the full epidemic.

For outbreaks seeded in Cumbria and simulated on the full dataset, we obtain a final mean epidemic size of 185 farms, with a mean duration of 22 weeks and a mean peak size of 45 farms (figure 2; electronic supplementary material, figure S8). The mean epidemic sizes after 6 and 12 weeks were 23 and 64 farms, respectively. For all node sampling methods without rescaling, the epidemic size is under-predicted as the percentage of nodes sampled decreases (figure 2). After six weeks, *S*_min_ = 3% for the TNS method, 20% for the SBS method and 80% for the RNS method. After 12 weeks, the percentage of nodes that must be sampled increases to 9%, 30% and 90% for the TNS, SBS and RNS methods, respectively (figure 2*b*). In order for these methods to accurately predict the full epidemic, 14%, 40% and 90% of the nodes must be sampled for the TNS, SBS and RNS methods, respectively (figure 2*c*). For the RMS method, *S*_min_ = 30% for 6 weeks, 50% for 12 weeks and 80% for the whole epidemic. Contour plots for epidemic size predictions for outbreaks seeded in Cumbria for each week of the outbreak (from week 1 to the end of the epidemic) are shown in electronic supplementary material, figures S8–S11. All methods provide accurate predictions of the size of the epidemic in the first few weeks. However, for longer durations, the TNS and the SBS methods provide the most robust predictions of epidemic size over time. Similar behaviour is observed for model predictions of epidemic duration and epidemic peak size (electronic supplementary material, figures S8–S11*b*,*c*)—the TNS method is able to accurately capture these characteristics when only 15% of the nodes are sampled, compared with 30%, 80% and 90% for the SBS, RMS and RNS methods, respectively.

The TNS method is consistently found to provide most accurate predictions of epidemic size, regardless of the county of disease introduction and disease parameters. In Devon, only 3% of the nodes require sampling for the TNS method to predict epidemic sizes at 6 weeks, compared with 10%, 20% and 80% for the SBS, RMS and RNS methods, respectively, with similar effects seen at 12 weeks and for the full epidemic (electronic supplementary material, figure S17). Similar behaviour is observed in Aberdeenshire (electronic supplementary material, figure S19). The values for *S*_min_ for the full epidemic for all sampling methods for epidemics seeded in the three counties are summarized in electronic supplementary material, table S1.

As the transmission rate of the disease increases, epidemic sizes increase and a higher percentage of nodes are required for all sampling methods to make accurate predictions. For example when *β* = 2, 25% of the nodes must be sampled using the TNS method and 50% for the SBS method to predict the overall epidemic size for outbreaks seeded in Cumbria, whereas for the RNS method almost all nodes must be sampled to capture epidemic behaviour (electronic supplementary material, figure S13). As the infectious period of the disease increases, a higher percentage of nodes needs to be sampled, but the effect of this is less pronounced than a variation in the transmission rate (electronic supplementary material, figures S14 and S15). For diseases with a very high transmission rate, a much higher percentage of nodes must be sampled for all methods, even when the infectious period is short (electronic supplementary material, figure S16).

When we include within-market transmission into our model, we observe significantly larger epidemic sizes, with the mean epidemic size after one infectious period when *β* = 1 and *T* = 21 being 2266 farms for outbreaks seeded in Cumbria. The TNS and SBS methods under-predict epidemic sizes when less than 35% and 50% respectively of the nodes are sampled (electronic supplementary material, figure S7). In contrast to the scenario where markets do not amplify transmission, the RMS method predicts epidemic sizes accurately even when only a very small number (approx. 20%) of movements are sampled. This suggests that, if a significant level of transmission is thought to occur within markets, then either TNS or RMS would be the preferred strategies if only limited resources were available. Similar results are observed for outbreaks seeded in Devon and Aberdeenshire.

### (c) Spatial spread

**Figure 3.**
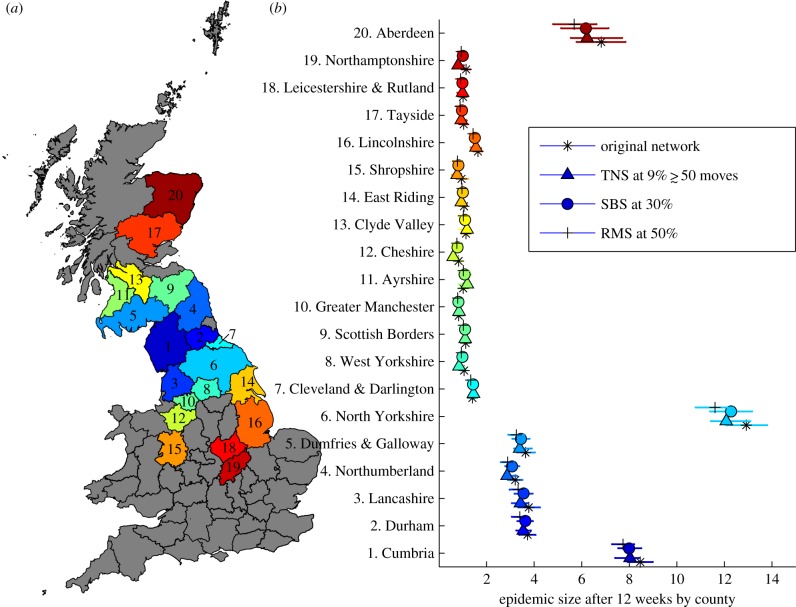
(*a*) A map of the 20 counties with the largest mean number of infected farms after 12 weeks when epidemics are seeded in Cumbria and markets are not explicitly included. (*b*) The average epidemic size for the original network (stars) random movement sampling (RMS) with 50% of sampled movements (crosses), snowball sampling with 30% of nodes (circles) and targeted node sampling (TNS), sampling nodes with more than 50 movements (triangles) for the 20 most infected counties when epidemics are seeded in Cumbria. Counties are ordered in terms of the proximity of their centroids from Cumbria.

It is important to consider not only the size of the simulated epidemics, but also how well the model captures the spatial spread of infection when partially sampled networks are used. When epidemics are seeded in Cumbria, almost all infected movements occur within Cumbria itself and to neighbouring counties (figure 3). An average of 8.9 farms become infected in Cumbria after 12 weeks, with 13.9 in North Yorkshire and 4.2, 4.0, 4.0 and 8.0 in Durham, Lancashire, Dumfries and Galloway, and Aberdeen, respectively. All other counties have epidemic sizes of fewer than two farms when the epidemic is seeded in Cumbria.

Using *S*_min_ for each of the sampling methods, we find that SBS captures the main epidemic hotspots well, but slightly overestimates epidemic sizes in these hotspots. RMS also performs well, but slightly underestimates epidemic sizes. The TNS method proves an accurate predictor of epidemic sizes in all the most infected counties, with 8.7 and 13.7 farms being infected on average after 12 weeks in Cumbria and North Yorkshire respectively (figure 3).

When markets are included the pattern of spatial spread is found to be similar to that without markets (electronic supplementary material, figure S12). The three most highly infected counties on the full network are Cumbria, North Yorkshire and Aberdeenshire, with mean epidemic sizes of 210, 258 and 238, respectively. When markets are included, we observe much larger epidemics in Devon, Somerset and northeast Wales. We also observe that each of the three sampling methods compares well with the original network at the *S*_min_ threshold.

Similar results are observed when outbreaks are seeded in Aberdeenshire and Devon—transmission within markets results in outbreaks with a much larger spatial extent than outbreaks in which markets do not play a role in transmission (electronic supplementary material, figures S21–S24).

## 4. Discussion

In order for models to be used to predict the potential for disease spread in livestock, there is reliance upon accurate data regarding farm locations and movements of livestock between farms. Significant work has been done in the UK to predict the potential for disease spread through the livestock network [12,17,18] owing to the existence of the CTS and the animal movement licence scheme. In many other countries around the world, the lack of such databases means that it is impossible to develop a model that uses precise movement data, and an alternative approach must be used. In such countries, it may be impossible to ever record all movement data either owing to the sheer size of the industry (in countries such as the USA) or owing to the cost associated with implementing an animal licence scheme. However, a more limited data collection scheme may be possible, whereby movements are recorded for a subset of the livestock movement network.

A simple way to collect a subset of livestock movement data would be to randomly sample all movements from a given set of random farms (i.e. using the RNS method). This method proved ineffective at reproducing the mean epidemics seen on the complete network. An alternative strategy to collect movement data would be to randomly sample movements from any farm (i.e. the RMS method). In a practical sense, this would be a much more difficult strategy to implement, requiring individual farmers to keep a record of livestock moving from their farm a given percentage of the time. This method is found to be more effective than the RNS method, particularly in the case when within-market transmission occurs. In that case, only 10–20% of movements are required in order to accurately predict epidemic sizes. For lower percentages, the model predicts smaller epidemics than observed using the true network data, and in that case suggested intervention strategies may not be sufficient to control outbreaks. It may, however, be possible to make accurate predictions with a lower percentage of movement data when incorporating a Bayesian kernel approach to scale up a partially observed network [43]. An alternative approach may be to adopt targeted movement sampling where movements would be recorded based on some criterion. For example, particularly frequent movements between pairs of nodes could be recorded, or shipments involving a large number of animals. Both these options were investigated, but neither proved to be particularly successful at reconstructing an accurate realization of the original network.

If only limited resources are available for data collection, it may be more efficient to record movements only from the most highly connected farms (the TNS method) or to use SBS (the SBS method). The TNS method proves significantly more effective than both the RMS and RNS methods when markets do not contribute to transmission—less than 20% of all farms would need to be surveyed in order to predict epidemic sizes to within 90% confidence in the UK for outbreaks seeded in Cumbria, Devon and Aberdeenshire. The model also gives a very good approximation of the spatial spread of the disease, the size of the epidemic peak and the epidemic duration. When markets contribute towards disease amplification, the TNS method requires that around 30% of all nodes would need to be surveyed in order to accurately predict epidemic sizes. The SBS method is found to perform less effectively than the TNS method, as this strategy rapidly identifies the most highly connected nodes that are likely to contribute most significantly to disease transmission. However, the SBS method may be more practical to implement as it does not require prior knowledge of the relative connectivity of the farms in the network.

The TNS and SBS methods have worked favourably in the livestock network described here. While one must take care when making inference from a subnetwork to the full network [28], it would be of great interest to the broader study of disease spread on partially observed networks to test these strategies further on livestock networks such as those available in other European countries [44–46]. The results of this work provide evidence of the viability of using partially sampled data to predict disease spread in livestock [21] and humans [23–26], and will inform data collection strategies in situations where complete knowledge of the network is impossible (e.g. wildlife movements [47,48]).

The role played by markets in disease transmission may have a significant effect upon the predictability of the sampling methods. When markets do not contribute to disease transmission, only a very small percentage of nodes needs to be sampled using the TNS method. However, when we make the assumption that all batches on a market are well mixed, a much larger proportion of the nodes must be sampled. We also find that in this case, the RMS method requires sampling of a much smaller percentage of movements than the non-market scenario. This is unsurprising—markets represent very highly connected nodes in the network, and therefore when they are explicitly included in the model, an RMS approach will preferentially sample movements to and from these highly connected nodes. The model currently assumes that livestock do not stay overnight on markets, in line with Defra policy, and hence any infectious markets would become susceptible the following day. Should this not be the case, the role of markets in disease transmission may be slightly altered. Therefore, our results suggest that a more thorough, disease-specific analysis of the precise role of markets in disease transmission would be required in the future in order to determine context-specific optimal sampling strategies. However, our sensitivity analysis shows that TNS is the preferred sampling strategy for all studied transmission rates and infectious periods.

The model presented in this paper uses a weighted static network to simulate the risk of transmission between livestock farms. Weighted static networks are regularly used in livestock disease models, and previous work indicates that they provide good prediction of mean epidemic sizes, though may potentially underestimate variability when compared with results on dynamic networks [38]. The advantage with a weighted static network approach is that it is possible to determine the epidemic impact independent of time of year. However, there is clear seasonality observed in the cattle movement network [49], and it is therefore possible that a weighted static network could result in an under- or over-prediction of epidemic size. Our sensitivity analysis suggests that the preferred sampling strategies are robust, although the proportion of nodes that need to be sampled may vary dependent upon time of year. Future studies will focus upon constructing a dynamic network, and testing network sampling schemes and temporal sampling schemes (whereby sampling is targeted based on time of year) on their ability to predict epidemic behaviour.

The results indicate that for a fast-spreading disease such as FMD, sampling a small proportion of the network is sufficient. This relies on the assumption that infected movements result in all livestock on the destination farm becoming rapidly infected. This is not the case for all livestock diseases. For example, animals infected with bTB can remain asymptomatic carriers for several months [13] before becoming infectious. The model framework described here would not be appropriate for a disease of this nature, and further work will focus upon the development of optimal sampling strategies for slow-spreading diseases such as bTB, where it may be crucial to track movements of individual cattle.

Our results suggest that for countries with similar farming practices, it may not be necessary to collect data on all livestock farms, but only those that contribute most significantly to the livestock trade. Of course, this creates something of a conundrum—in order to sample the most highly connected nodes, and thus accurately represent epidemic risk on an unknown network, one needs to know which farms have the most movements. One solution to this would be for all farmers to be required to record the number of movements they make in a given year. These summary statistics could then be used to determine which farms should be sampled for the following year. In the UK, at least, analysis of the movement network for multiple years suggests that those farms that have a high number of movements in a given year are more likely to have a high number of movements in the following year. This method may therefore be used in countries where livestock movement data are not currently available in order to inform epidemic models and predict the potential for disease spread owing to animal movements in the early stages of a disease outbreak.

## Supplementary Material

Equivalence of rebuilding methods

## Supplementary Material

Supplementray figures SI 1 - SI 5

## Supplementary Material

Supplementray figures SI 6 - SI 24

## Supplementary Material

Algorithms
