# Supplementary material for: Epidemic predictions in an imperfect world: modelling disease spread with partial data
Source: Proc Biol Sci. 2015 Jun 7;282(1808):20150205. doi: 10.1098/rspb.2015.0205 (PMC4455802; doi:10.1098/rspb.2015.0205)
Supplement: Equivalence of rebuilding methods [file rspb20150205supp1.pdf]

Supporting Information

Equivalence of rebuilding by scaling with rebuilding by preferentially increasing edge weight

In this paper we utilize a simple re-scaling method to rebuild partially sampled networks. An apparently more sophisticated approach would be to adapt the weighted scale free model approach of Yook *et al.*[1] to a static network where new edges are not created but edge weights are increased preferentially based upon the weights of existing edges. However, we can show that these two methods are, in fact, equivalent.

Given that the depleted network begins with some fraction α of the original number of movements N_0 and we wish to add n movements such that $N_0 = \alpha N_0 + n$ where the probability of movement from farm i to j being added is given by

$$p(i \to j,t) = \frac{a_{ij}(t)}{\sum a_{ij}(t)} = \frac{N(i \to j,t)}{\alpha N_0 + t}.$$

The weight of the edge between i and j at time $t+1$ is then

$$\begin{aligned} N(i \to j,t+1) &= N(i \to j,t) + p(i \to j,t) \\ &= N(i \to j) + \frac{N(i \to j,t)}{\alpha N_0 + t} \end{aligned}$$

if we then enforce the initial condition that the depleted network begins with N_D movements, that is

$$N(i \to j,0) = N_D(i \to j)$$

this can be solved explicitly as

$$\begin{aligned} N(i \to j,n) &= N_D(i \to j) \prod_{t=1}^{n-1} \left(1 + \frac{1}{\alpha N_0 + t}\right) \\ &= N_D(i \to j) \frac{n + \alpha N_0}{1 + \alpha N_0} \\ &= N_D(i \to j) \frac{N_0}{1 + \alpha N_0} \end{aligned}$$

and assuming that $\alpha N_0 >> 1$ which it is, we obtain the the result

$$N_n(i \to j) = \frac{1}{\alpha} N_D(i \to j). \tag{3}$$

Therefore if we were to carry out the preferential weighting method many times the average weights would be the same as the re-scaling method we utilize in this paper.

[1] Yook S, Jeong H, Barabási AL, and Tu Y (2001), Weighted evolving networks, *Phys Rev Lett*, 86(25):5835–5838.
