# Supplementary material for: Epidemic predictions in an imperfect world: modelling disease spread with partial data
Source: Proc Biol Sci. 2015 Jun 7;282(1808):20150205. doi: 10.1098/rspb.2015.0205 (PMC4455802; doi:10.1098/rspb.2015.0205)
Supplement: Supplementray figures SI 1 - SI 5 [file rspb20150205supp2.pdf]

Supplementary figures: Networks

Assortativity is the tendency for similar nodes to connect to each other in a network, and is measured using the Pearson correlation function for the node attribute under inspection, generally the node degree. The assortativity value ranges from -1 for a totally dissortative network to +1 for a totally assortative network. If the correlation is zero, there is no tendency for nodes with a similar degree to connect or actively avoid each other. In the case of a directed network there are four types of degree correlation to consider, i) r_{in-in}, the correlation between the in-degree of the incident node and the in-degree of the target node, ii) r_{in-out}, the correlation between the in-degree of the incident node and the out-degree of the target node, iii) $r_{out-out}$, the correlation between the out-degree of the incident node and the out-degree of the target node and iv) r_{out-in}, the correlation between the out-degree of the the incident node and the in-degree of the target node. For a full and detailed explanation of these measures and a discussion on their implications see Foster *et al* 2010 [40]. For the node sampling schemes described in the text the degree correlations are plotted in figure SI 4 with r_{in-in} being plotted for the movement sampling scheme in figure SI 5 f).

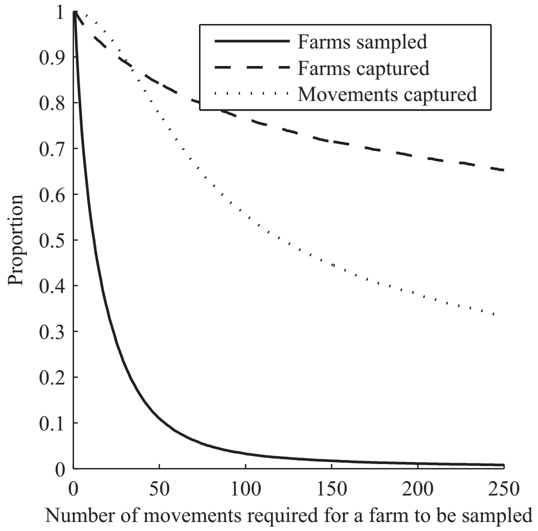

Figure SI 1: Plot showing the proportion of farms with at least x number of movements (solid line). The proportion of farms (dashed line) and movements (dotted line) captured when a targeted node sampling scheme is used to sample the farms with at least x number of movements (solid line) is also shown.

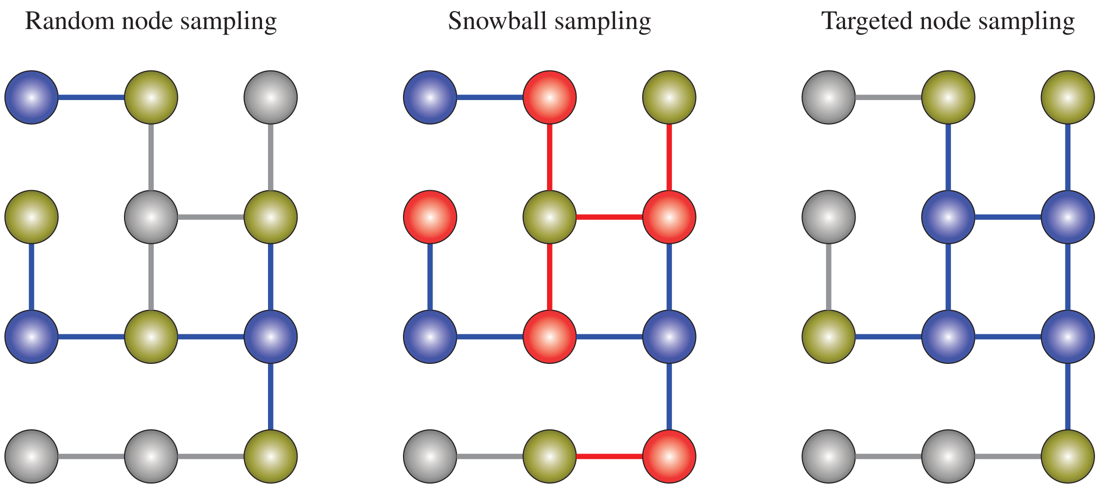

Figure SI 2: Schematic illustration showing the three sampling schemes employed. Sampled nodes and edges are in blue for initially sampled nodes and red for the case of second stage snowball sampling (centre). Nodes discovered by the sampled nodes (captured-nodes) are coloured green with their non-sampled edges in grey. Nodes which have not been sampled or discovered are coloured grey. Here second stage snowball sampling is depicted, at the third stage all green nodes would turn red, their edges would turn red and gray nodes connected to them would turn green. For targeted node sampling (right) node with degree three or higher have been sampled.

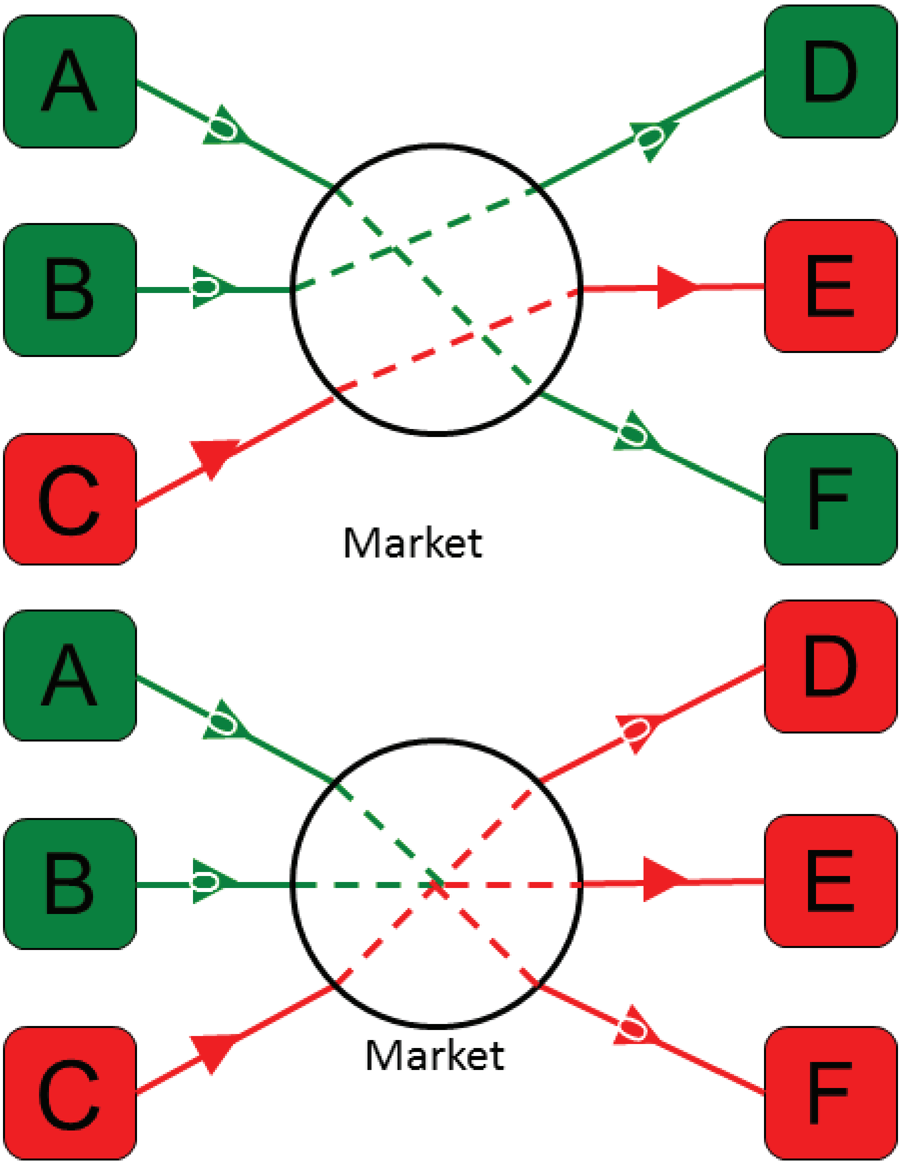

Figure SI 3: Two extremes of markets are included in this paper. In the first instance bio-security on a market is effectively assumed to be perfect, markets play no role in the transmission process. This is depicted in the upper figure where an infected movement (red) from farm C passes through the market but the only farm with a probability of being infected is its target farm, farm E. In the other extreme we assume that market have effectively no bio-security and infected movement from farm E has potential to infect the market, if it does so all farms receiving movements from the market have a non-zero probability of becoming infected.

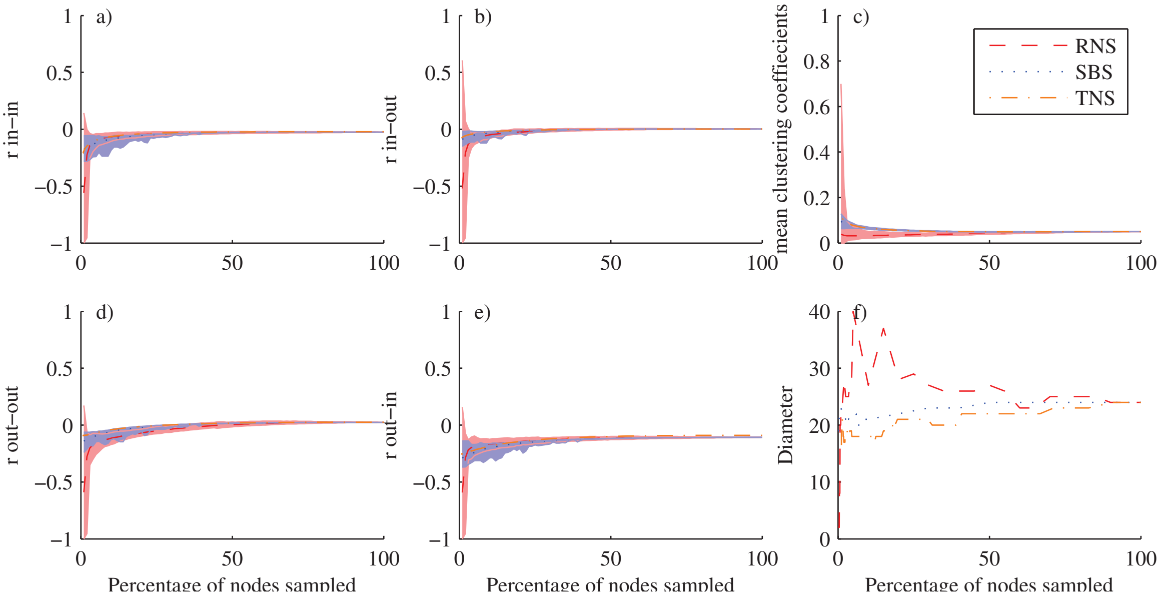

Figure SI 4: (Graphs (a), (b), (d) and (e) show the directed degree-degree correlations for the three node sampling measures as the percentage of nodes sampled varies. The assortativity measures are defined using the Pearson correlation explained in the text above. The mean local clustering coefficient is plotted in (c). The shaded regions on plots (a) to (e) indicate the maximum and minimum values obtained for these measured over 1000 realisations of the networks for RNS (dashed line), SBS (dotted line) and TNS (dot-dashed line). Plot f) shows the network diameter for one realisation of the network for each of the sampling schemes.

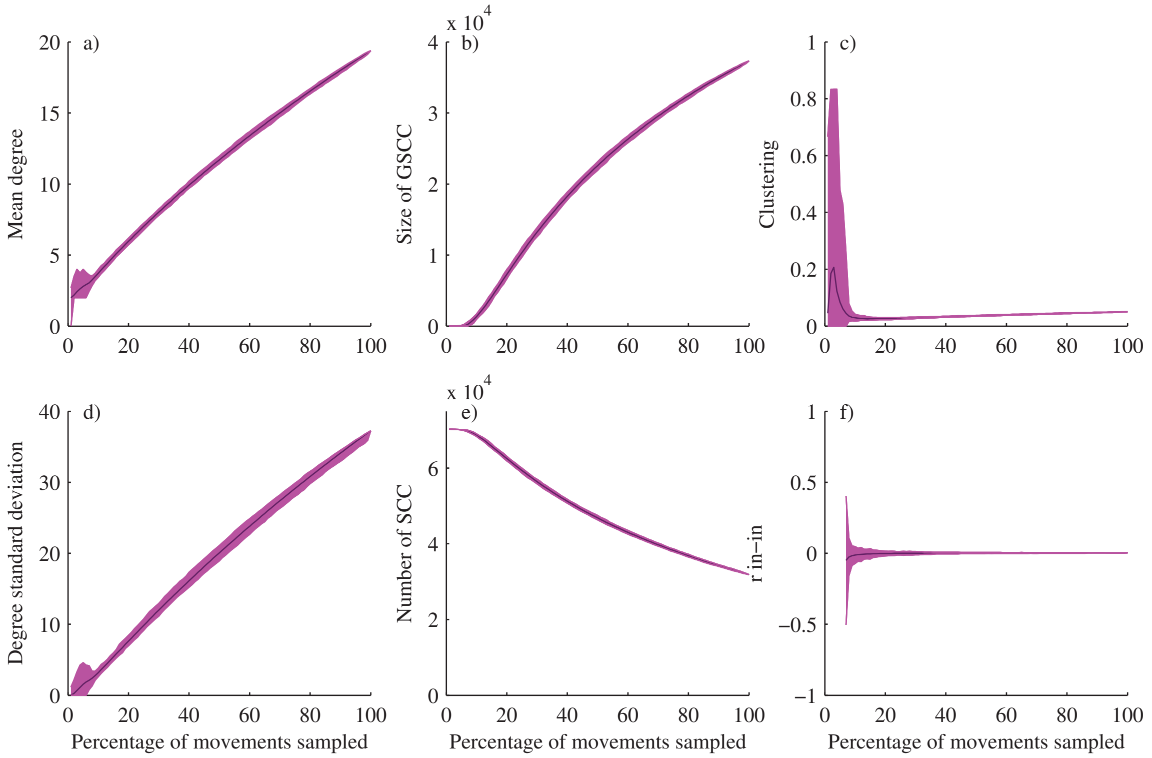

Figure SI 5: Graphs showing (a) the mean degree, (b) the size of the giant strongly connected component, (c), the mean local clustering coefficient,(d) the degree standard deviation, (e) the number of strongly connected components and (f) the correlation for for the RMS method as a function of the percentage of movements sampled. These statistics are averaged over 1000 realisations with shaded confidence intervals depicting the maximum and minimum value of each statistic.
