# Supplementary material for: Epidemic predictions in an imperfect world: modelling disease spread with partial data
Source: Proc Biol Sci. 2015 Jun 7;282(1808):20150205. doi: 10.1098/rspb.2015.0205 (PMC4455802; doi:10.1098/rspb.2015.0205)
Supplement: Supplementray figures SI 6 - SI 24 [file rspb20150205supp3.pdf]

Overview of the minimum sampling threshold for outbreaks seeded in Devon, Cumbria and Aberdeenshire for all sampling methods

S_{min}	Cumbria	Devon	Aberdeen
RMS without markets	50%	40%	60%
SBS without markets	30%	20%	30%
TNS without markets	9% (50)	3% (80)	8% (60)
RMS with markets	20%	10%	10%
SBS with markets	50%	40%	50%
TNS with markets	30% (20)	25% (25)	30% (20)

Table 1: Summary of the minimum sampling threshold S_{min} after 12 weeks for random movement sampling (RMS), snowball node sampling (SBS) and targeted node sampling (TNS) without markets and S_{min} after one infectious period with markets for epidemics seeded in Cumbria, Devon and Aberdeen. Parameters were $\beta = 1$ and $T = 21$ days. For TNS the number of movements a farm needs in order top be sampled is given in parentheses.

Supplementary figures for Cumbria $\beta = 1$, $T = 21$ days

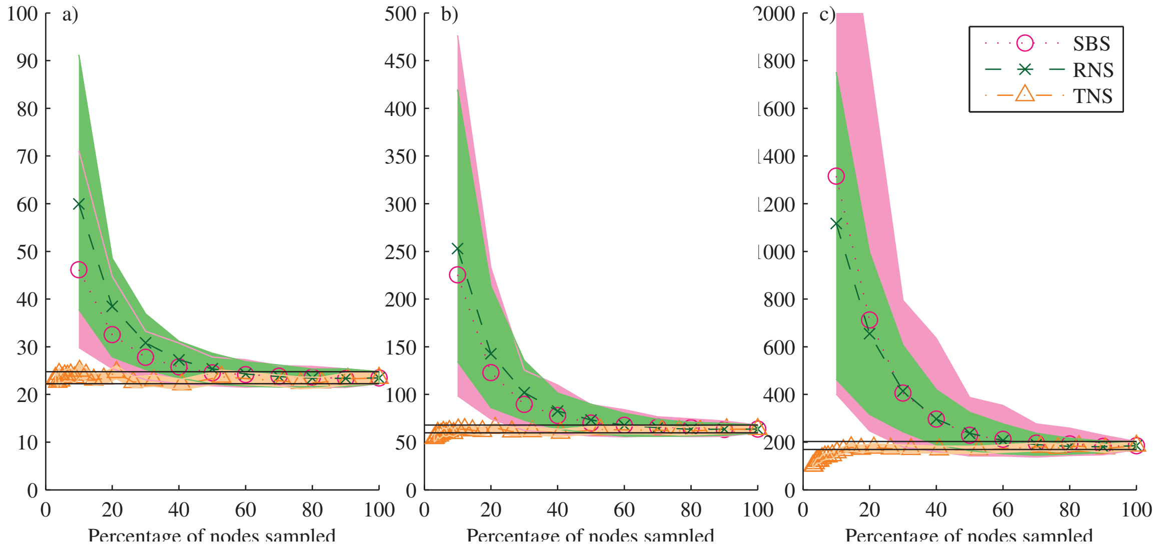

Figure SI 6: Epidemic measurements for outbreaks seeded in Cumbria using the RNS (crosses) method, SBS (circles) method and the TNS (triangles) method with shaded 95% confidence intervals, when $\beta = 1$ and $T = 21$ days for (a) 6 weeks, (b) 12 weeks and (c) the whole epidemic. In this plot, the RNS and SBS methods are scaled as in equation (1).

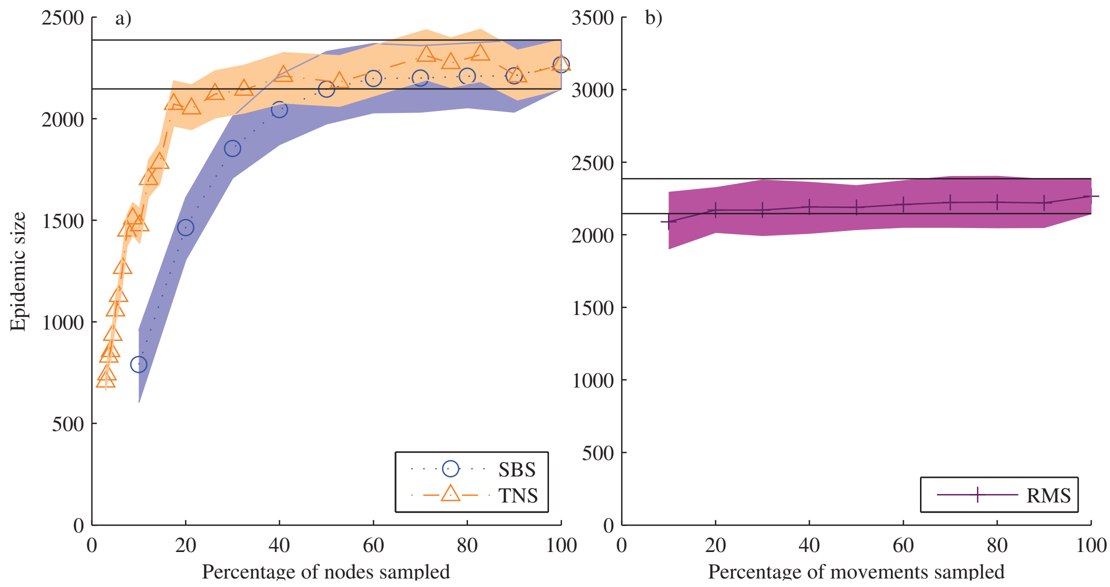

Figure SI 7: Mean epidemic size for outbreaks seeded in Cumbria, with within-market transmission incorporated into the model when $\beta = 1$ and $T = 21$, for (a) the TNS (triangles) and the SBS (circles) methods and (b) the RMS (crosses) method. Shaded 95% confidence intervals are shown in each figure for each sampling method.

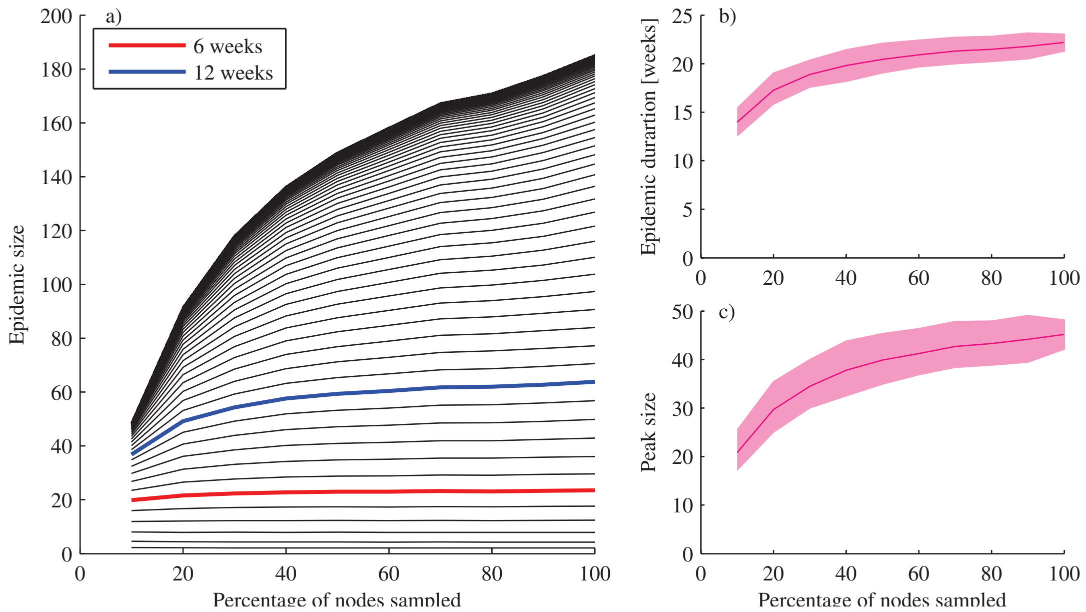

Figure SI 8: Epidemic measurements for outbreaks seeded in Cumbria using the RMS method with $\beta = 1$ and $T = 21$ days. Plot a) shows the mean epidemic size in weekly intervals with ascending contours representing the epidemic size progression through time in weeks, the size after 6 (red) and 12 (blue) weeks are highlighted for reference. Plot b) shows the mean epidemic duration in weeks with 95% confidence intervals. Plot c) shows the mean peak size of the epidemic with 95% confidence intervals.

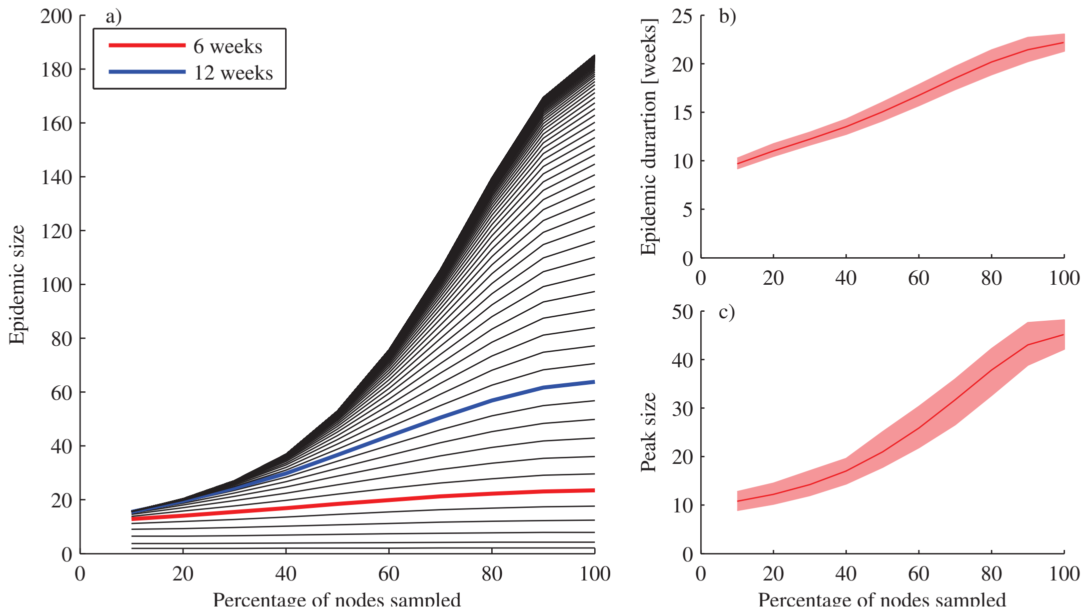

Figure SI 9: Epidemic measurements for outbreaks seeded in Cumbria using the RNS method with $\beta = 1$ and $T = 21$ days. Plot a) shows the mean epidemic size in weekly intervals with ascending contours representing the epidemic size progression through time in weeks, the size after 6 (red) and 12 (blue) weeks are highlighted for reference. Plot b) shows the mean epidemic duration in weeks with 95% confidence intervals. Plot c) shows the mean peak size of the epidemic with 95% confidence intervals.

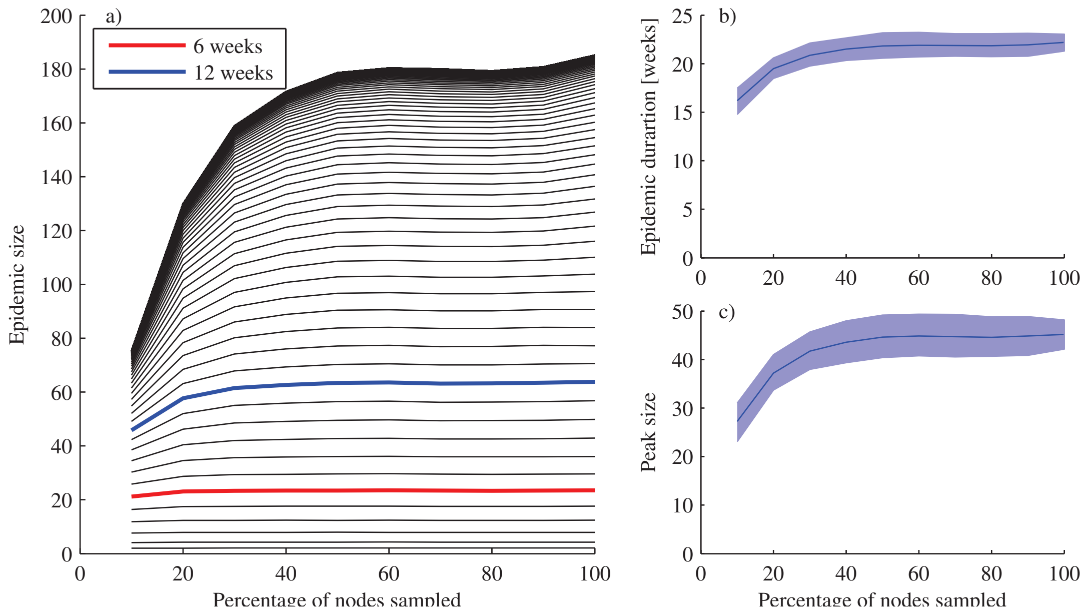

Figure SI 10: Epidemic measurements for outbreaks seeded in Cumbria using the SBS method with $\beta = 1$ and $T = 21$ days. Plot a) shows the mean epidemic size in weekly intervals with ascending contours representing the epidemic size progression through time in weeks, the size after 6 (red) and 12 (blue) weeks are highlighted for reference. Plot b) shows the mean epidemic duration in weeks with 95% confidence intervals. Plot c) shows the mean peak size of the epidemic with 95% confidence intervals.

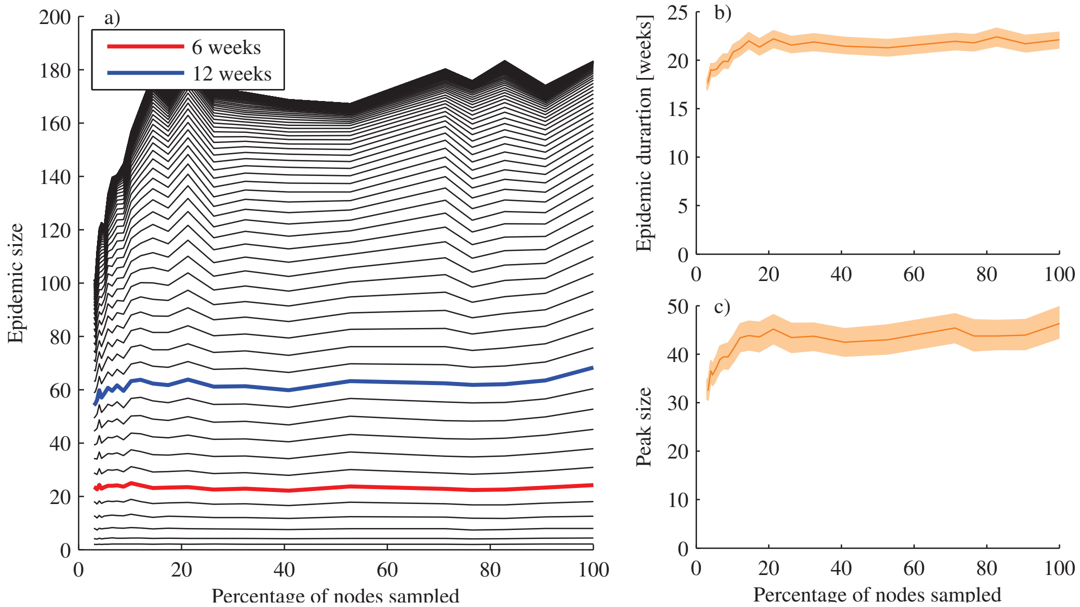

Figure SI 11: Epidemic measurements for outbreaks seeded in Cumbria using the TNS method with $\beta = 1$ and $T = 21$ days. Plot a) shows the mean epidemic size in weekly intervals with ascending contours representing the epidemic size progression through time in weeks, the size after 6 (red) and 12 (blue) weeks are highlighted for reference. Plot b) shows the mean epidemic duration in weeks with 95% confidence intervals. Plot c) shows the mean peak size of the epidemic with 95% confidence intervals.

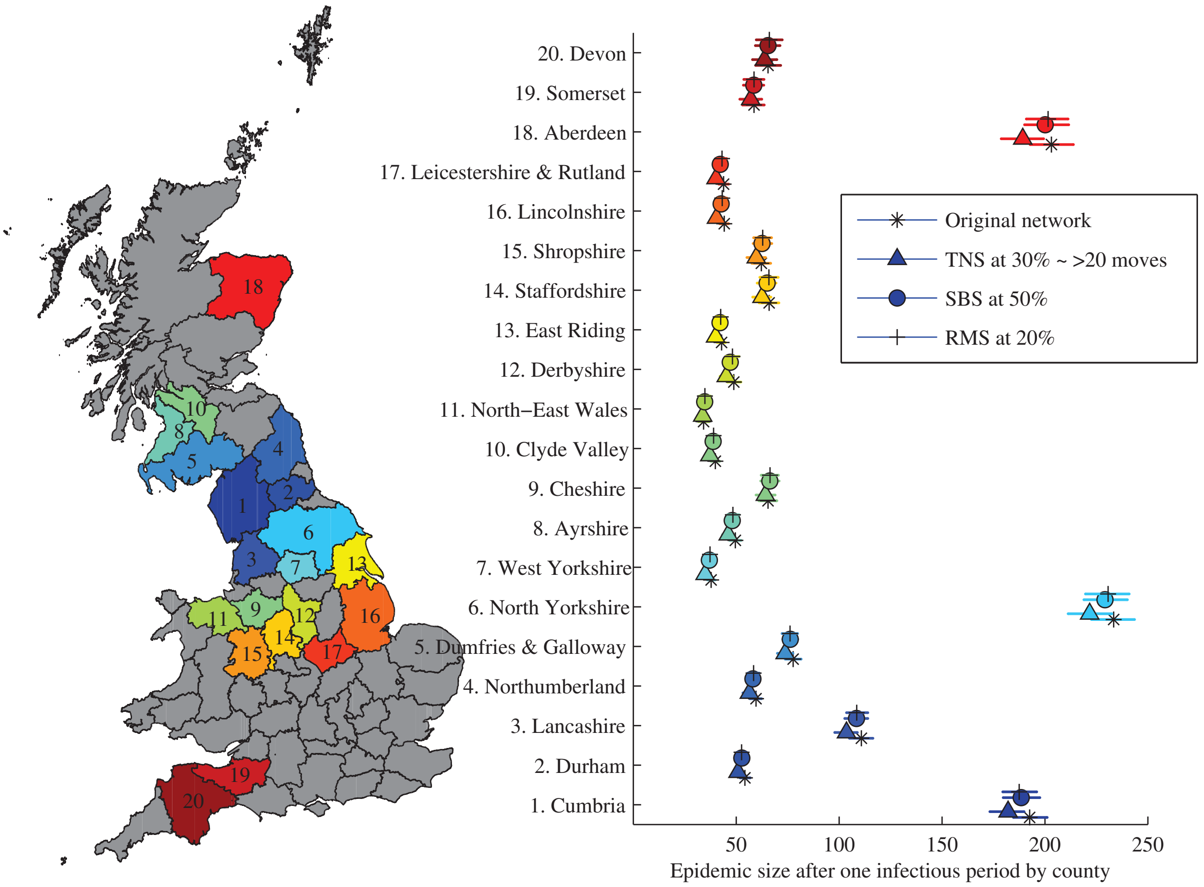

Figure SI 12: The left panel shows a map of the 20 counties with the largest mean number of infected farms after one infectious period when epidemics are seeded in Cumbria and markets are included, $\beta = 1$ and $T = 21$. The right panel shows the average epidemic size for the original network (stars) random movement sampling (RMS) with 20% of sampled movements (crosses), snowball sampling with 50% of nodes (circles) and targeted node sampling (TNS), sampling nodes with more than 20 movements (triangles) for the 20 most infected counties when epidemics are seeded in Cumbria. Counties are ordered in terms of the proximity of their centroids from Cumbria.).

Supplementary figures for Cumbria with a range of parameters

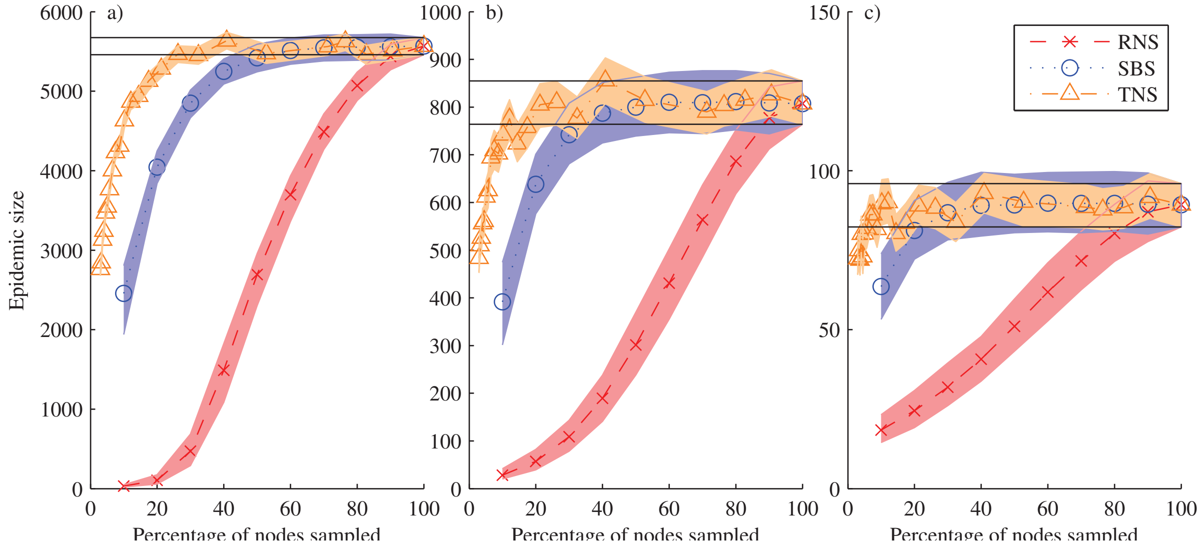

Figure SI 13: Epidemic measurements for outbreaks seeded in Cumbria using the RNS (crosses), SBS (circles) and TNS (triangles) methods with $\beta = 2$ and $T = 21$ days. Plot a) shows the final epidemic size, plot b) the epidemic size after 12 weeks and plot c) the epidemic size after 6 weeks. Shaded 95% confidence intervals are included as are black reference lines indicating the 95% confidence intervals for simulations run on the complete network.

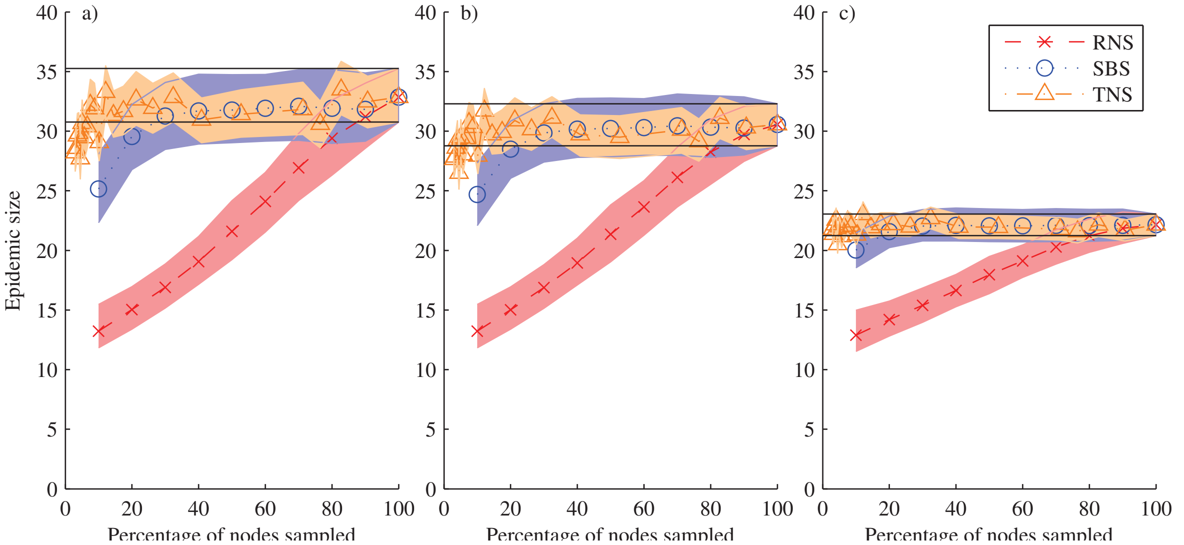

Figure SI 14: Epidemic measurements for outbreaks seeded in Cumbria using the RNS (crosses), SBS (circles) and TNS (triangles) methods with $\beta = 1$ and $T = 14$ days. Plot a) shows the final epidemic size, plot b) the epidemic size after 12 weeks and plot c) the epidemic size after 6 weeks. Shaded 95% confidence intervals are included as are black reference lines indicating the 95% confidence intervals for simulations run on the complete network.

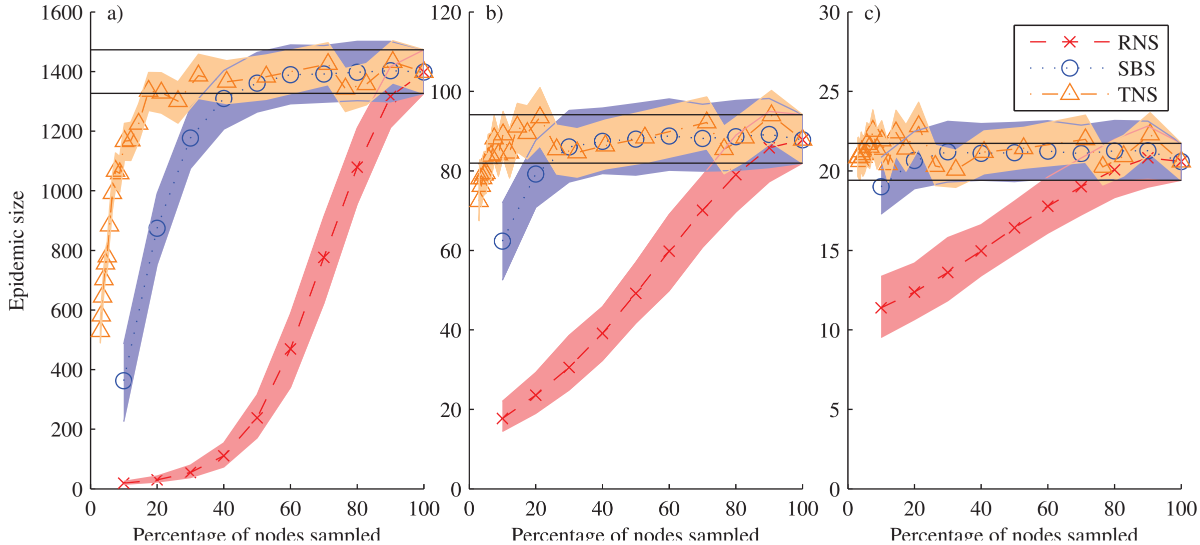

Figure SI 15: Epidemic measurements for outbreaks seeded in Cumbria using the RNS (crosses), SBS (circles) and TNS (triangles) methods with $\beta = 1$ and $T = 28$ days. Plot a) shows the final epidemic size, plot b) the epidemic size after 12 weeks and plot c) the epidemic size after 6 weeks. Shaded 95% confidence intervals are included as are black reference lines indicating the 95% confidence intervals for simulations run on the complete network.

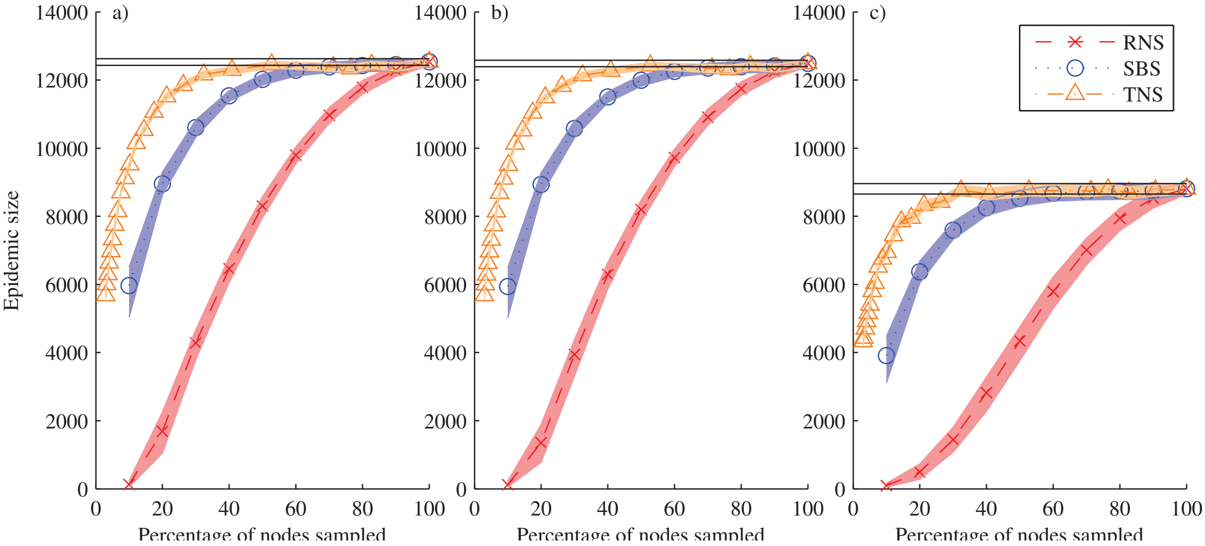

Figure SI 16: Epidemic measurements for outbreaks seeded in Cumbria using the RNS (crosses), SBS (circles) and TNS (triangles) methods with $\beta = 10$ and $T = 7$ days. Plot a) shows the final epidemic size, plot b) the epidemic size after 12 weeks and plot c) the epidemic size after 6 weeks. Shaded 95% confidence intervals are included as are black reference lines indicating the 95% confidence intervals for simulations run on the complete network.

Supplementary figures for epidemics seeded in Aberdeenshire and Devon

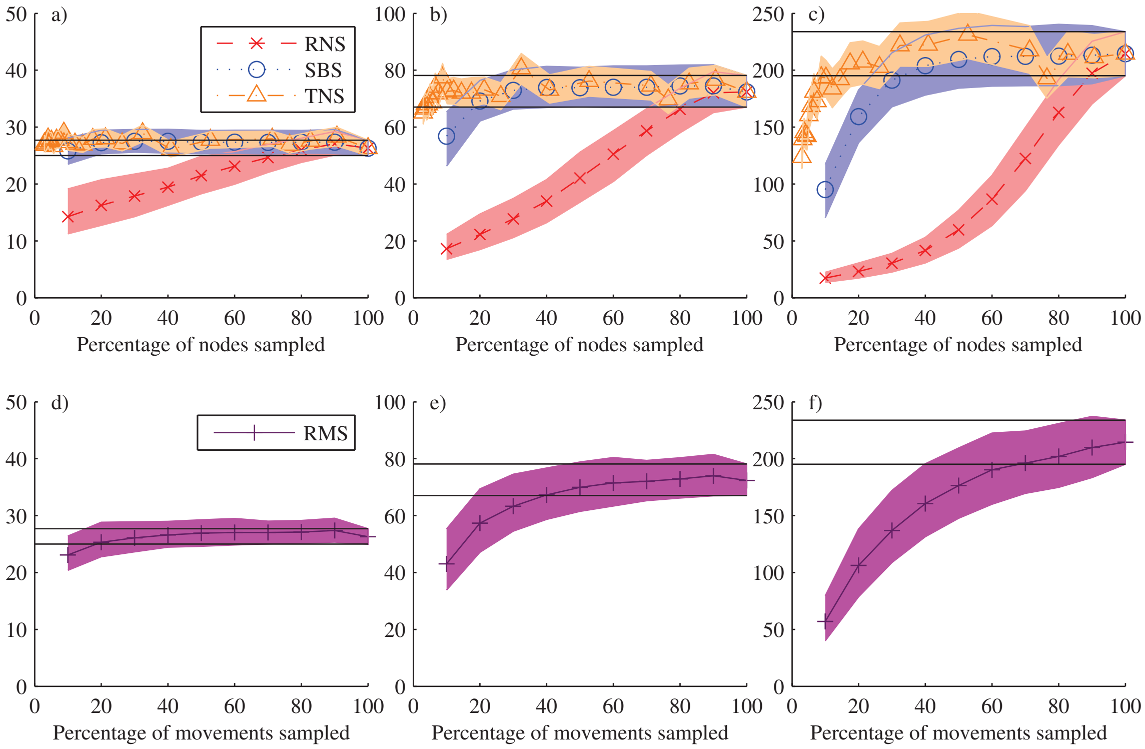

Figure SI 17: Graphs (a) to (c) compare the epidemic size for outbreaks seeded in Devon on networks generated by RNS (crosses), SBS (circles) and TNS (triangles) as a function of nodes sampled with shaded 95% confidence intervals for (a) 6 weeks, (b) 12 weeks and (c) the full epidemic. The solid black lines represents the 95% confidence intervals on the average simulation for the original network. Graphs (d) to (f) show the same results for the RMS method for (d) 6 weeks, (e) 12 weeks and (f) the full epidemic.

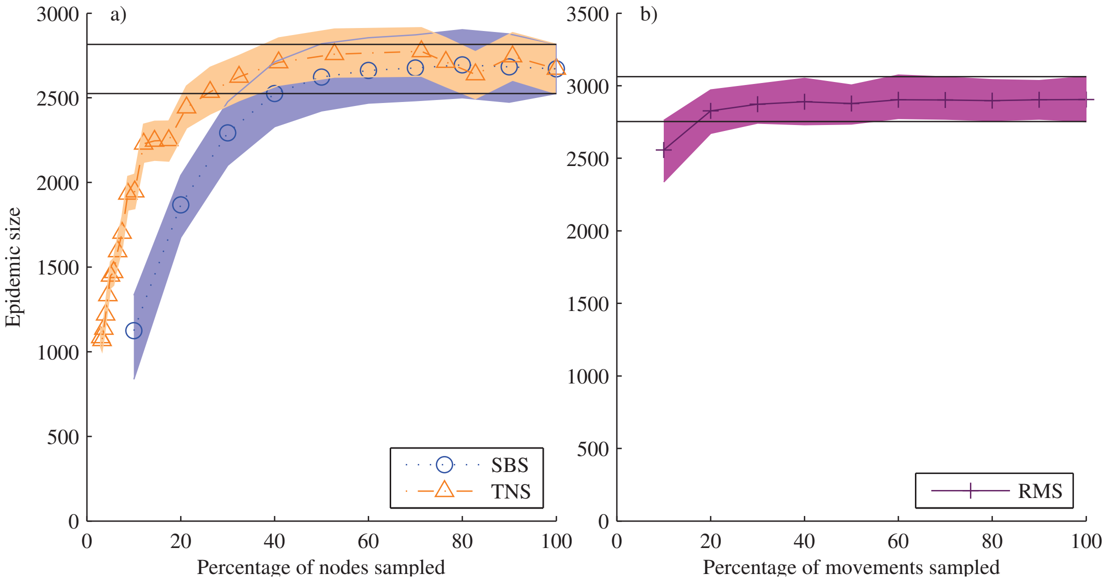

Figure SI 18: Mean epidemic size for outbreaks seeded in Devon, with within-market transmission incorporated into the model when $\beta = 1$ and $T = 21$, for (a) the TNS (triangles) and the SBS (circles) methods and (b) the RMS (crosses) method. Shaded 95% confidence intervals are shown in each figure for each sampling method.

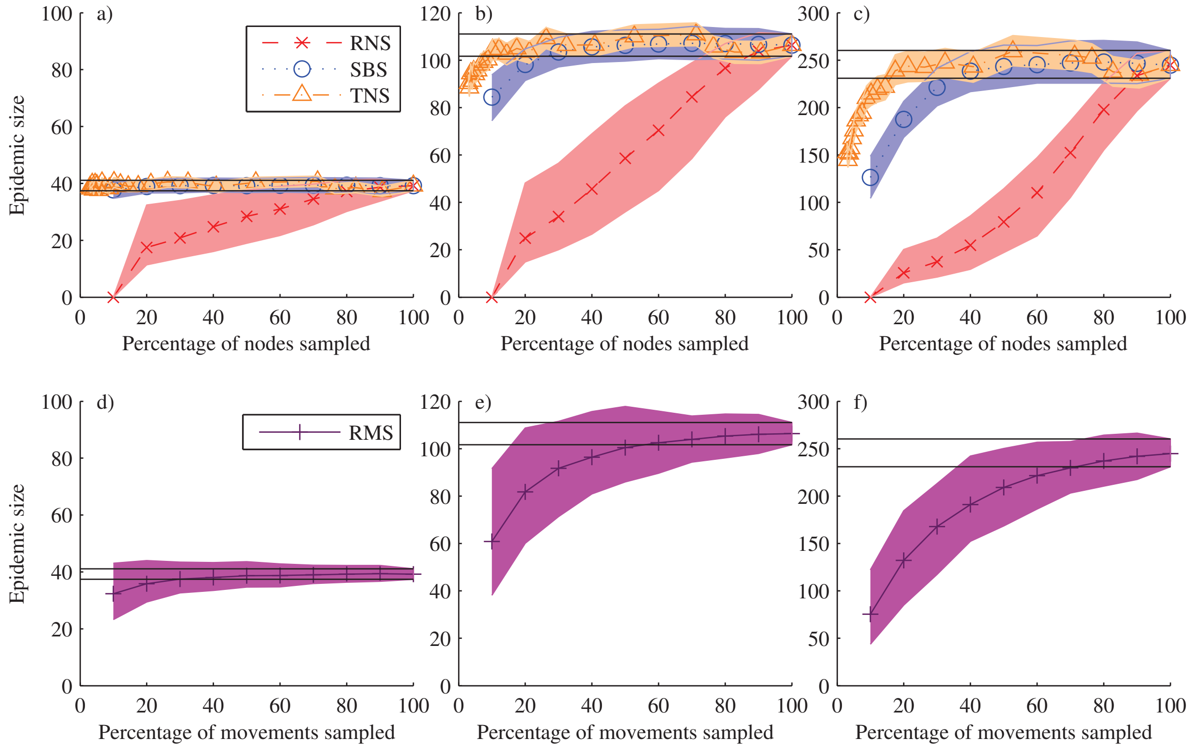

Figure SI 19: Graphs (a) to (c) compare the epidemic size for outbreaks seeded in Aberdeen on networks generated by RNS (crosses), SBS (circles) and TNS (triangles) as a function of nodes sampled with shaded 95% confidence intervals for (a) 6 weeks, (b) 12 weeks and (c) the full epidemic. The solid black lines represents the 95% confidence intervals on the average simulation for the original network. Graphs (d) to (f) show the same results for the RMS method for (d) 6 weeks, (e) 12 weeks and (f) the full epidemic.

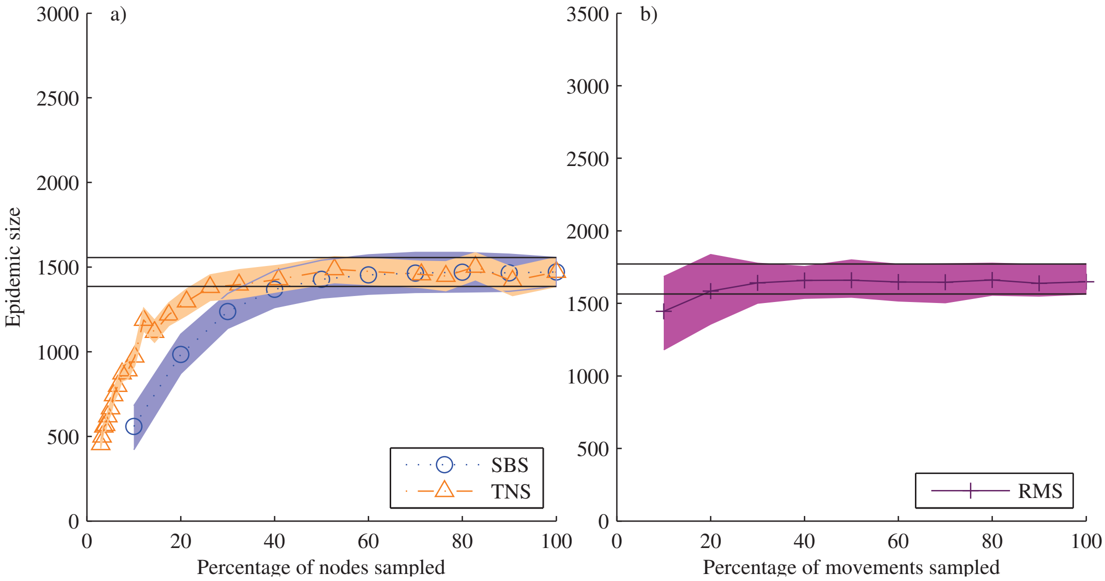

Figure SI 20: Mean epidemic size for outbreaks seeded in Aberdeen, with within-market transmission incorporated into the model when $\beta = 1$ and $T = 21$, for (a) the TNS (triangles) and the SBS (circles) methods and (b) the RMS (crosses) method. Shaded 95% confidence intervals are shown in each figure for each sampling method.

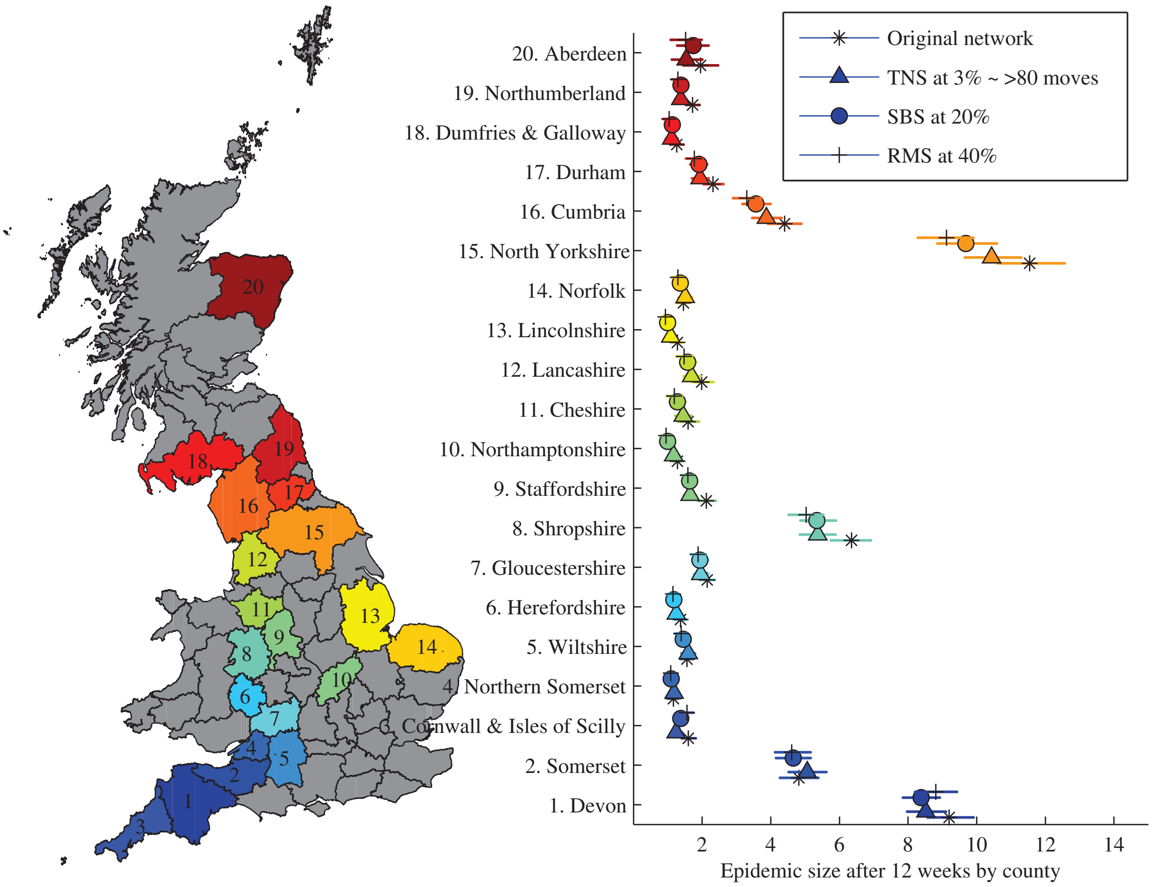

Figure SI 21: The left panel shows a map of the 20 counties with the largest mean number of infected farms after 12 weeks when epidemics are seeded in Devon and markets are not explicitly included. The right panel shows the average epidemic size for the original network (stars) random movement sampling (RMS) with 40% of sampled movements (crosses), snowball sampling with 20% of nodes (circles) and targeted node sampling (TNS), sampling nodes with more than 80 movements (triangles) for the 20 most infected counties when epidemics are seeded in Cumbria. Counties are ordered in terms of the proximity of their centroids from Devon.).

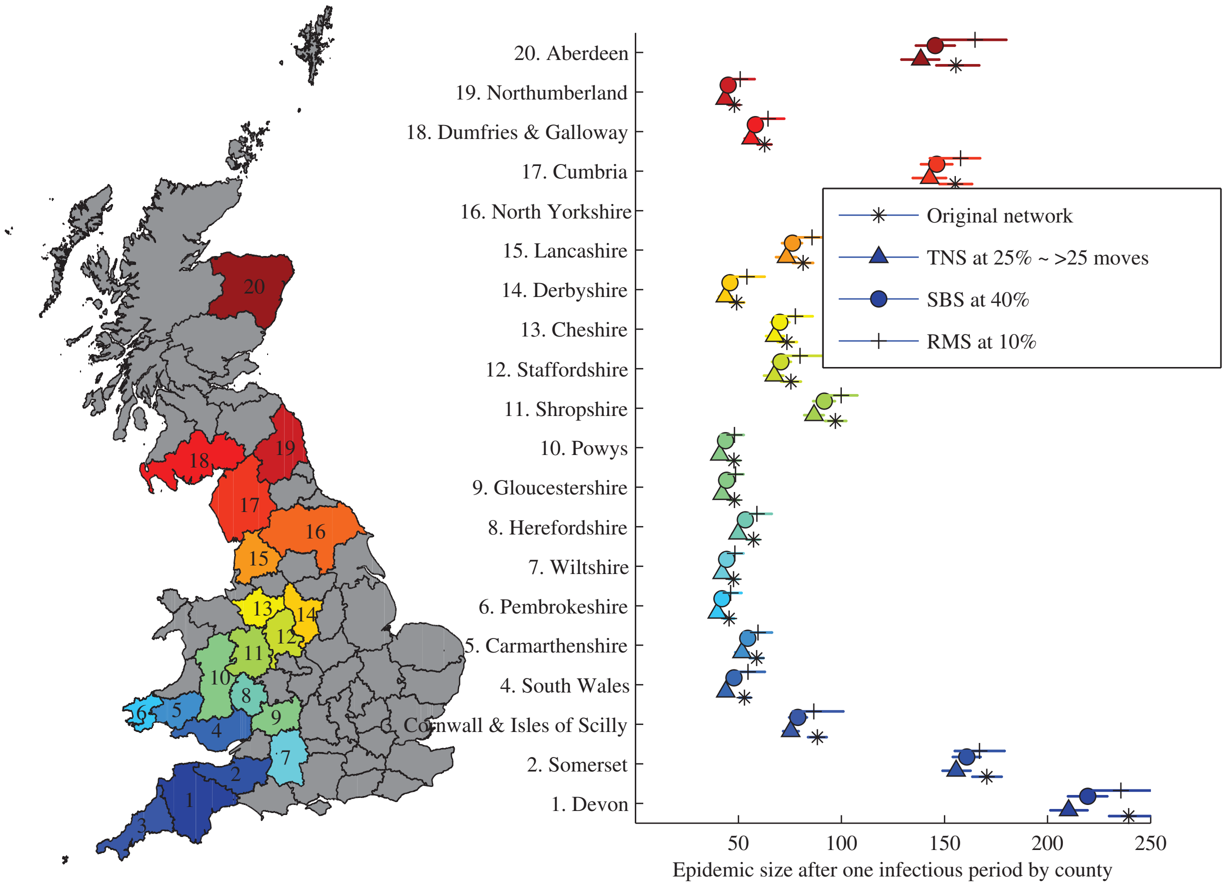

Figure SI 22: The left panel shows a map of the 20 counties with the largest mean number of infected farms after one infectious period when epidemics are seeded in Devon and markets are included, $\beta = 1$ and $T = 21$. The right panel shows the average epidemic size for the original network (stars) random movement sampling (RMS) with 10% of sampled movements (crosses), snowball sampling with 40% of nodes (circles) and targeted node sampling (TNS), sampling nodes with more than 25 movements (triangles) for the 20 most infected counties when epidemics are seeded in Cumbria. Counties are ordered in terms of the proximity of their centroids from Cumbria.).

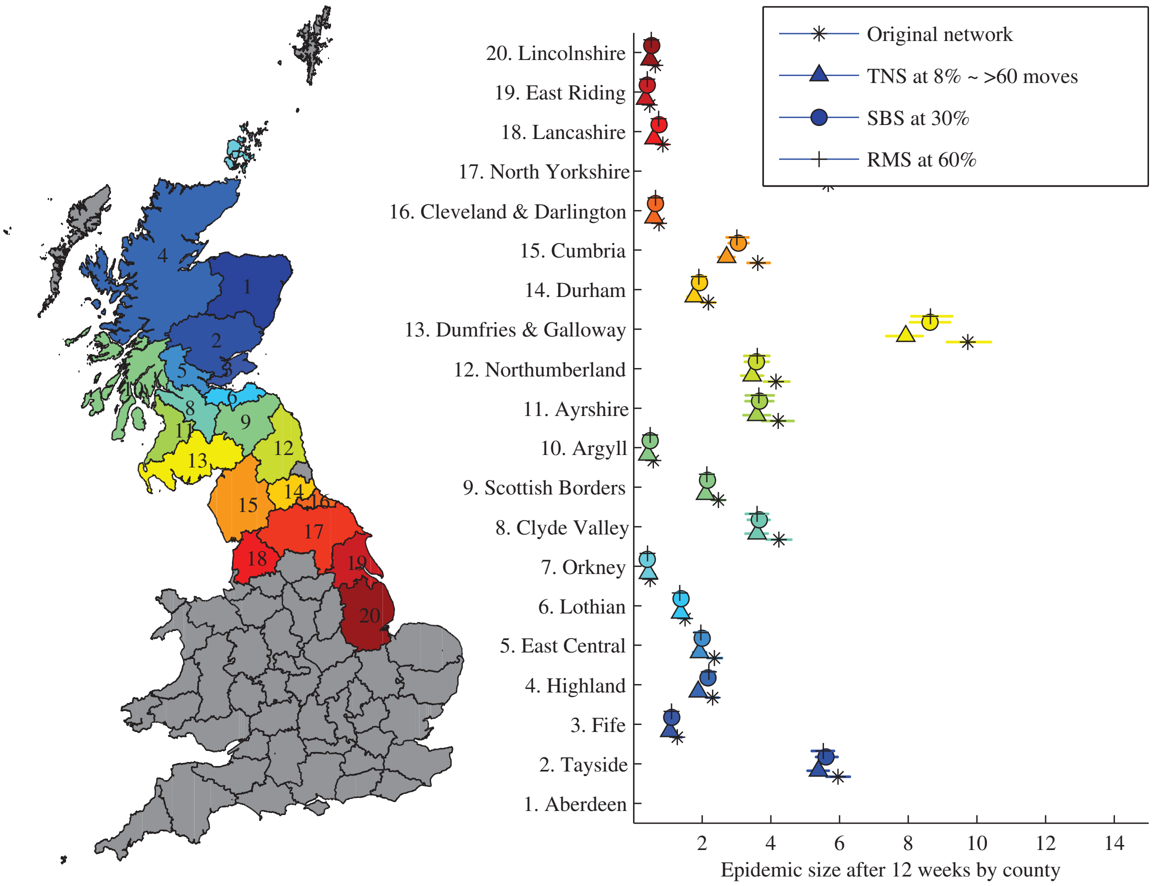

Figure SI 23: The left panel shows a map of the 20 counties with the largest mean number of infected farms when epidemics are seeded in Aberdeen and markets are not explicitly included, $\beta = 1$, $T = 21$ days. The right panel shows the average epidemic size for the original network (circles,) random movement sampling (RMS) with 40% of sampled movements (triangles),random node sampling with 55% of nodes (squares) and targeted node sampling (TNS), sampling nodes with more than 54 movements (diamonds) for the 20 most infected counties when epidemics are seeded in Devon. Counties are ordered in terms of the proximity of their centroids from Devon.).

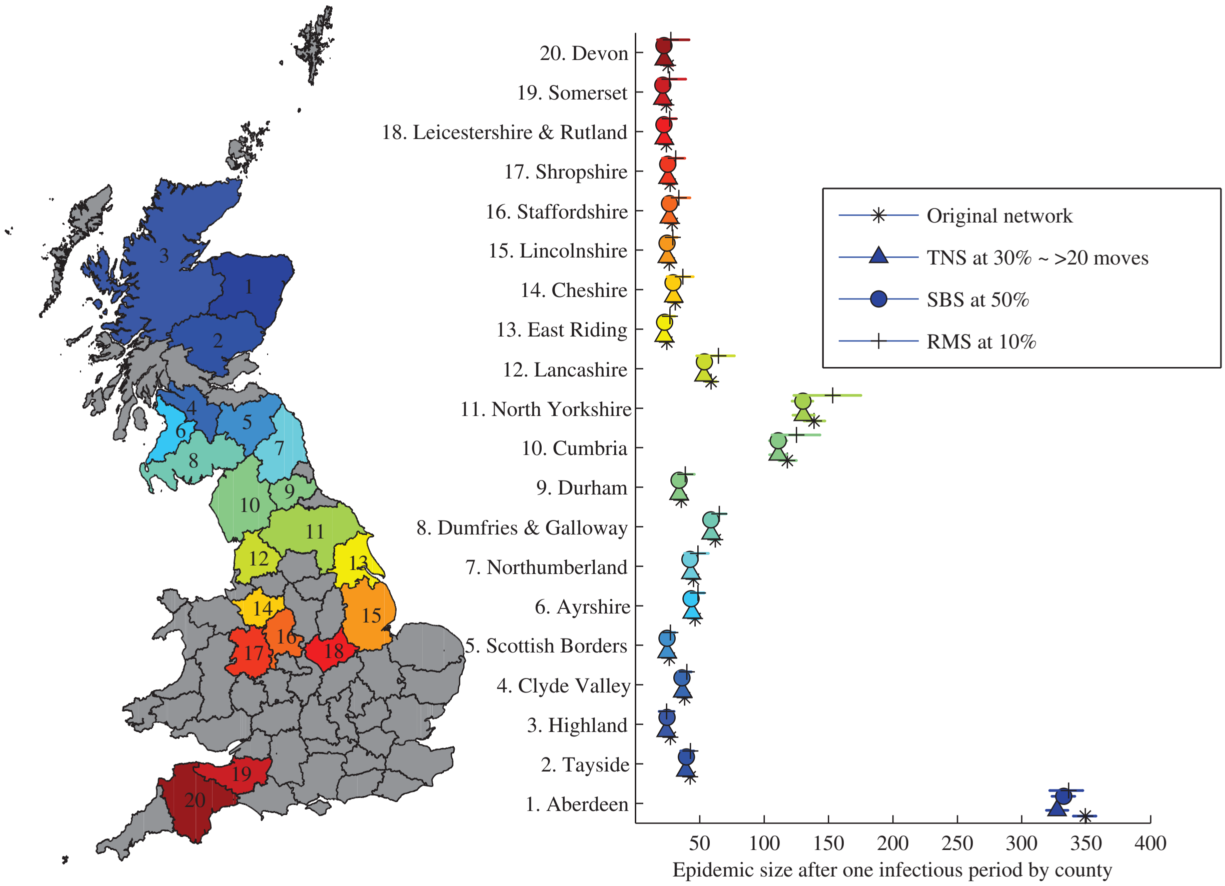

Figure SI 24: The left panel shows a map of the 20 counties with the largest mean number of infected farms after 12 weeks when epidemics are seeded in Aberdeen and markets are not explicitly included. The right panel shows the average epidemic size for the original network (stars) random movement sampling (RMS) with 10% of sampled movements (crosses), snowball sampling with 50% of nodes (circles) and targeted node sampling (TNS), sampling nodes with more than 50 movements (triangles) for the 20 most infected counties when epidemics are seeded in Cumbria. Counties are ordered in terms of the proximity of their centroids from Aberdeen.).

Algorithm 1 Standard SIR

$t = 1$;
$I(k) = 1$; ▷ Seed farm k to initially infected
$I_{\text{time}} = 1$ ▷ Record time of infection
while $\sum(I) > 0$ **do**
    for $i = 1 : N$ **do** ▷ Infection loop
        Calculate λ_i from equation 2
        $p = RAND$
        if $\lambda_i < p$ **then**
            $I(i) = 1$
            $I_{\text{time}} = t$
        end if
    end for
    for $i = 1 : N$ **do** ▷ Recovery loop
        if $I_{\text{time}} - T = 0$ **then**
            $R(i) = 0$
        end if
    end for
    $t{+}{+}$
end while

Algorithm 2 Epidemic process including markets with an SIS model

while $\sum(I) > 0$ **do**
    Infection loop for markets
    Infection loop for farms
    Recovery loop for farms
    Set all markets to be susceptible
    $t{+}{+}$
end while
