# Supplementary material for: Epidemic predictions in an imperfect world: modelling disease spread with partial data
Source: Proc Biol Sci. 2015 Jun 7;282(1808):20150205. doi: 10.1098/rspb.2015.0205 (PMC4455802; doi:10.1098/rspb.2015.0205)
Supplement: Algorithms [file rspb20150205supp4.pdf]

Algorithm 1 Standard SIR

$t = 1$;
$I(k) = 1$; ▷ Seed farm k to initially infected
$I_{\text{time}} = 1$ ▷ Record time of infection
while $\sum(I) > 0$ **do**
  for $i = 1 : N$ **do** ▷ Infection loop
    Calculate λ_i from equation 2
    $p = RAND$
    if $\lambda_i < p$ **then**
      $I(i) = 1$
      $I_{\text{time}} = t$
    end if
  end for
  for $i = 1 : N$ **do** ▷ Recovery loop
    if $I_{\text{time}} - T = 0$ **then**
      $R(i) = 0$
    end if
  end for
  $t{+}{+}$
end while

Algorithm 2 Epidemic process including markets with an SIS model

while $\sum(I) > 0$ **do**
  Infection loop for markets
  Infection loop for farms
  Recovery loop for farms
  Set all markets to be susceptible
  $t{+}{+}$
end while
